# Co-benefits from sustainable dietary shifts for population and environmental health: an assessment from a large European cohort study

**DOI:** 10.1016/S2542-5196(21)00250-3

**Published:** 2021-10-22

**Authors:** Jessica E Laine, Inge Huybrechts, Marc J Gunter, Pietro Ferrari, Elisabete Weiderpass, Kostas Tsilidis, Dagfinn Aune, Matthias B Schulze, Manuela Bergmann, Elisabeth H M Temme, Jolanda M A Boer, Claudia Agnoli, Ulrika Ericson, Anna Stubbendorff, Daniel B Ibsen, Christina Catherine Dahm, Mélanie Deschasaux, Mathilde Touvier, Emmanuelle Kesse-Guyot, Maria-Jose Sánchez Pérez, Miguel Rodríguez Barranco, Tammy Y N Tong, Keren Papier, Anika Knuppel, Marie-Christine Boutron-Ruault, Francesca Mancini, Gianluca Severi, Bernard Srour, Tilman Kühn, Giovanna Masala, Antonio Agudo, Guri Skeie, Charlotta Rylander, Torkjel M Sandanger, Elio Riboli, Paolo Vineis

**Affiliations:** a MRC Centre for Environment and Health, School of Public Health, Imperial College London, London, UK; b Institute for Social and Preventive Medicine, University of Bern, Bern, Switzerland; c Department of Epidemiology and Biostatistics, Faculty of Medicine, School of Public Health, Imperial College London, London, UK; d International Agency for Research on Cancer, Lyon, France; e Department of Nutrition, Bjørknes University College, Oslo, Norway; f Department of Endocrinology, Morbid Obesity and Preventive Medicine, Oslo University Hospital, Ullevål, Oslo, Norway; g Department of Molecular Epidemiology, German Institute of Human Nutrition Potsdam-Rehbruecke, Nuthetal, Germany; h Institute of Nutritional Sciences, University of Potsdam, Nuthetal, Germany; i Centre for Nutrition, Prevention and Health Services National Institute for Public Health and the Environment (RIVM), Bilthoven, Netherlands; j Epidemiology and Prevention Unit Fondazione IRCCS Istituto Nazionale dei Tumori di Milano, Milano, Italy; k Department of Clinical Sciences, Lund University, Malmö, Sweden; l Research Unit for Epidemiology, Department of Public Health, Aarhus University, Aarhus, Denmark; m Sorbonne Paris Nord University, Inserm U1153, Inrae U1125, Cnam, Nutritional Epidemiology Research Team (EREN), Epidemiology and Statistics Research Center – University of Paris (CRESS), Bobigny, France; n Escuela Andaluza de Salud Pública (EASP), Granada, Spain; o Instituto de Investigación Biosanitaria ibs.GRANADA, Granada, Spain; p Centro de Investigación Biomédica en Red de Epidemiología y Salud Pública (CIBERESP), Madrid, Spain; q Department of Preventive Medicine and Public Health, University of Granada, Granada, Spain; r Cancer Epidemiology Unit, Nuffield Department of Population Health, University of Oxford, Oxford, UK; s CESP, Faculté de médecine, Université Paris-Saclay, UVSQ, INSERM, 94805, Villejuif, France; t Gustave Roussy, F-94805, Villejuif, France; u Department of Statistics, Computer Science and Applications “G. Parenti”, University of Florence, Florence, Italy; v Division of Cancer Epidemiology, German Cancer Rsearch Center Heidelberg, Heidelberg, Germany; w Cancer Risk Factors and Life-Style Epidemiology Unit, Institute for Cancer Research, Prevention and Clinical Network - ISPRO, Florence, Italy; x Unit of Nutrition and Cancer, Catalan Institute of Oncology - ICO, Nutrition and Cancer Group, Bellvitge Biomedical Research Institute (IDIBELL), L'Hospitalet de Llobregat, Barcelona, Spain; y Department of Community Medicine, UiT the Arctic University of Norway, Tromsø, Norway; z Italian Institute of Technology, Genova, Italy

## Abstract

**Background:**

Unhealthy diets, the rise of non-communicable diseases, and the declining health of the planet are highly intertwined, where food production and consumption are major drivers of increases in greenhouse gas emissions, substantial land use, and adverse health such as cancer and mortality. To assess the potential co-benefits from shifting to more sustainable diets, we aimed to investigate the associations of dietary greenhouse gas emissions and land use with all-cause and cause-specific mortality and cancer incidence rates.

**Methods:**

Using data from 443 991 participants in the European Prospective Investigation into Cancer and Nutrition (EPIC) study, a multicentre prospective cohort, we estimated associations between dietary contributions to greenhouse gas emissions and land use and all-cause and cause-specific mortality and incident cancers using Cox proportional hazards regression models. The main exposures were modelled as quartiles. Co-benefits, encompassing the potential effects of alternative diets on all-cause mortality and cancer and potential reductions in greenhouse gas emissions and land use, were estimated with counterfactual attributable fraction intervention models, simulating potential effects of dietary shifts based on the EAT–*Lancet* reference diet.

**Findings:**

In the pooled analysis, there was an association between levels of dietary greenhouse gas emissions and all-cause mortality (adjusted hazard ratio [HR] 1·13 [95% CI 1·10–1·16]) and between land use and all-cause mortality (1·18 [1·15–1·21]) when comparing the fourth quartile to the first quartile. Similar associations were observed for cause-specific mortality. Associations were also observed between all-cause cancer incidence rates and greenhouse gas emissions, when comparing the fourth quartile to the first quartile (adjusted HR 1·11 [95% CI 1·09–1·14]) and between all-cause cancer incidence rates and land use (1·13 [1·10–1·15]); however, estimates differed by cancer type. Through counterfactual attributable fraction modelling of shifts in levels of adherence to the EAT–*Lancet* diet, we estimated that up to 19–63% of deaths and up to 10–39% of cancers could be prevented, in a 20-year risk period, by different levels of adherence to the EAT–*Lancet* reference diet. Additionally, switching from lower adherence to the EAT–*Lancet* reference diet to higher adherence could potentially reduce food-associated greenhouse gas emissions up to 50% and land use up to 62%.

**Interpretation:**

Our results indicate that shifts towards universally sustainable diets could lead to co-benefits, such as minimising diet-related greenhouse gas emissions and land use, reducing the environmental footprint, aiding in climate change mitigation, and improving population health.

**Funding:**

European Commission (DG-SANCO), the International Agency for Research on Cancer (IARC), MRC Early Career Fellowship (MR/M501669/1).

## Introduction

Diets are a key link between population health and environmental sustainability, as unhealthy diets, the rise of non-communicable diseases, and the declining health of the planet are highly intertwined. Across several populations, access to and consumption of micronutrient-rich foods, such as fresh fruits, vegetables, legumes, and so on, are largely inadequate, whereas access to and consumption of high-calorie and processed foods high in salt, sugars, saturated and trans fats, and foods from animal sources are now more widely available.^1^ Shifts in the underlying food system over the past 50 years have contributed to nutrition transitions, where the rise in obesity and non-communicable diseases, including type 2 diabetes, cancers, cardiovascular diseases, and early mortality has become a global public health crisis.^2^ Food production is also a major driver of global environmental footprints, including increases in greenhouse gas emissions and substantial water and land use, among others, intensifying climate change and environmental degradation. For example, agriculture, with a large contribution from livestock production, is responsible for up to 25% of anthropogenic greenhouse gas emissions and approximately 70% of freshwater use, and uses more than a third of cultivable land.^3^ However, there are opportunities to reduce these impacts by changing diets, food production, and food systems.

**Research in context**

**Evidence before this study**

Although no formal literature search, such as a systematic review, was done before undertaking this study, the available evidence indicates that the declining health of the planet and humans can be mitigated by altering unsustainable diets and food systems, resulting in co-benefits. Although previous studies have estimated potential co-benefits of altering diets to mitigate environmental impacts and improve population health, there are many unknowns and uncertainties that need to be addressed to inform both health and environmental policies. Specifically, previous studies have mostly used aggregated data at the country level on diet-related contributions to poorer health (eg, mortality) and environmental impacts of dietary patterns (eg, greenhouse gas emissions), diets have been typically assessed at a macro level of large groups of food items (eg, meats *vs* vegetables) with little individual dietary information, and usually only one environmental footprint has been assessed at a time (ie, greenhouse gas reduction). Additionally, co-benefits that meet both environmental standards (eg, reduction of greenhouse gas emissions) and the complexities of nutritional requirements have not been holistically examined in a manner applicable across populations and with cohort data. Last, the potential of shifting diets to achieve co-benefits has not been assessed with interventional models that consider counterfactual scenarios. Addressing these limitations is crucial to determine the role of diets in producing co-benefits to improve population and planetary health.

**Added value of this study**

This study is, to our knowledge, the largest co-benefits assessment based on prospective cohort data (comprising >400 000 participants) from the European Prospective Investigation into Cancer and Nutrition (EPIC) study, with a follow-up of 14 years. We estimated the health impacts for all-cause and cause-specific mortality and cancer rates from greenhouse gas emissions and land use using detailed dietary information from more than 11 000 food items and identified the impact on the health and the environment by adopting a sustainable alternative diet, the EAT–*Lancet* diet, by simulating interventions. We found associations between levels of greenhouse gas emissions and land use (estimated from dietary components) and all-cause and cause-specific mortality, all cancers, and some organ-specific cancers. By simulating a dietary intervention that considers nutritional needs and environmental footprints, the EAT–*Lancet* diet, we found that all-cause mortality and all cancers could be substantially reduced, together with reductions in greenhouse gas emissions and land use.

**Implications of all the available evidence**

Our findings, along with other studies, including the EAT–*Lancet* report, suggest that co-benefits to human health and the environment could be achieved by adopting diets that consider both nutritional quality and planetary impact, such as the EAT–*Lancet* diet. Addressing dietary patterns that represent co-benefits could help in climate change mitigation, in addition to reducing other sources of greenhouse gas emissions, and might help reduce the incidence of diet-related mortality and cancers.

Diets that improve both the health of people and the environment have been deemed as a win-win situation, leading to co-benefits, where mitigation of dietary-related greenhouse gas emissions and land use, among other environmental impacts, could improve population health (eg, by reducing early mortality) and the environment (eg, by reducing the rate of climate change). Although research on the potential co-benefits of various diets is widespread, it has largely been limited to assessing either the impacts of dietary factors on the environment or the effects of altering diets on health, with very few studies incorporating the two.^4^ Additionally, many studies measure co-benefits with aggregated data at the country level, using modelled data, and typically diets are assessed at a macro level of large groups of food items (eg, meats *vs* vegetables) with little detailed dietary information at an individual level. Several alternative diets studied for their potential co-benefits include ones that focus on altering specific nutrients (eg, micronutrient consumption),^5^ reducing meat consumption,^6^ adhering to a Mediterranean diet,^7^ or comparing diets such as Mediterranean, pescatarian, and vegetarian diets,^8^ and those that consider different dietary scenarios with reduced greenhouse gas emissions or other environmental targets.^3^, ^9^ However, many of these suggested diets do not holistically address both the need to meet environmental standards (eg, reduction of greenhouse gas emissions) and the complexities of nutritional requirements, nor are they applicable across populations. Additionally, findings for the potential to achieve co-benefits from shifting diets have been inconsistent.^5^ Recently, a reference diet was proposed by the EAT–*Lancet* Commission to meet targets for a global food system to promote human and environmental health.^10^ The association between a score derived from the EAT–*Lancet* diet and major health outcomes was evaluated in the European Prospective Investigation into Cancer and Nutrition (EPIC) Oxford centre (largely in vegetarians), where adherence to a higher score seemed to be beneficial for some, but not all, health outcomes.^11^

Understanding the environmental impact of diets on both population health and planetary health is crucial for the development of sustainable public health policies and for improving planetary health. To address the population-level health implications of dietary contributions to higher environmental footprints and the potential co-benefits from shifting to alternative, more sustainable diets, we aimed to investigate the associations of dietary greenhouse gas emissions and land use with health outcomes of all-cause and cause-specific mortality and cancer incidence rates. Additionally, we used counterfactual intervention models to simulate the effects of dietary interventions to determine the co-benefits of alternative diets. Our estimates are based on individual food frequency questionnaire data from a large population.

## Methods

### Cohort description

EPIC, a multicentre prospective cohort study, was designed to investigate the relationship between nutrition and cancer, among other diseases. A detailed description of the EPIC cohort, including study populations and data collection, has been previously described elsewhere,^12^ and is provided in more detail for the present study in the appendix (pp 1–2). Briefly, EPIC consists of 23 study centres in ten European countries: Denmark, France, Germany, Greece, Italy, the Netherlands, Norway, Spain, Sweden, and the UK. Participants were mostly from the general population and recruited between 1991 and 2000. Diet was assessed at study baseline by use of validated country-specific or centre-specific methods, including dietary questionnaires spanning the previous 12 months.^12^ Cause-specific mortality data were coded according to the 10th revision of the International Statistical Classification of Diseases, Injuries and Causes of Death (ICD-10) (appendix p 1), and include coronary heart disease, cardiovascular disease, cancer, and respiratory disease. Incident cancer cases were identified through several methods, including record linkage with population-based cancer registries, health insurance records, pathology registries, autopsy or death certificates, and active follow-up of study participants. Some participants with missing information were excluded from the present study (appendix p 1). The final dataset comprised 443 991 participants. Any additional missing data on demographics (eg, education, smoking, and physical activity) were imputed with the R package mice.^13^

### Greenhouse gas emissions and land use calculations from food frequency data

Greenhouse gas emissions and land use were estimated from detailed standardised country-specific dietary questionnaires, with the SHARP-Indicators Database, a European-wide database for estimating environmental impacts of food production, packaging, transport, and home preparation.^14^ The total food list for EPIC comprised 11 858 food items. Specific food items were matched (appendix pp 2–3, 5–7) between the EPIC database and the SHARP database, based on their FoodEx2 code from the Exposure Hierarchy of the European Food Safety Authority.^15^ Greenhouse gas emissions were expressed as kg CO_2_ equivalents per kg food per day and land use as m^2^ per year per kg food per day.

### Posited causal structure and potential confounding variables

The main exposures considered in relation to the outcomes of all-cause and cause-specific mortality and cancer are greenhouse gas emissions and land use levels derived from individual diets, modelled as quartiles of 0–25% (1st quartile as the reference quartile), more than 25% up to 50% (2nd quartile), more than 50% up to 75% (3rd quartile), and more than 75% (4th quartile). A set of confounders was selected on the basis of their potential association with the exposure and outcome, and if they were not known to be on the causal pathway based on a directed acyclic graph. However, because the causal pathways of the exposure–outcome associations considered are not well known, several sensitivity analyses of potential residual confounding were done and are described in the appendix (p 3). All models were adjusted for the following set of potential confounders: age at recruitment (continuous), marital status (dichotomised as not married or married or living together), education (categorised into four: not educated to primary school, technical or professional school, high school, or higher education at university level), physical activity (dichotomised as active or not active), smoking status (trichotomised as never, or former or current smoker), and body-mass index (BMI; continuous). Because EPIC is a multicentre study, designed with prospective harmonisation in mind, our main analyses considered the entire EPIC cohort, where data were pooled. Pooled models were adjusted for the potential confounders listed above and additionally for country as a fixed effect. Country-specific models were also run to assess the heterogeneity of estimates among cohorts.

### Association of greenhouse gas emissions and land use with mortality

To estimate the association of greenhouse gas emissions and land use with all-cause and cause-specific mortality, hazard ratios (HRs) and 95% CIs were estimated. For all-cause mortality, Cox proportional hazards regression models were run. For cause-specific models, assessing coronary heart disease, cardiovascular disease, cancer, and respiratory disease, competing risk models were run, accounting for each cause of mortality separately with the R package riskRegression. This method uses a binomial regression model based on a time sequence of binary event status variable. Competing risk models were considered as participants could have experienced one or more events, thus competing for the outcome of mortality, whereby not doing so might not appropriately estimate the cumulative incidence when competing events are censored.^16^ For both all-cause and cause-specific mortality models, the underlying time scale considered was person-years from the start of the study until the date of death, date of emigration, loss to follow-up, or end of follow-up, whichever occurred first. Differential follow-up was accounted for in the competing risks model. Adjusted HRs were estimated, adjusting for the set of potential confounders listed above. Restricted cubic spline modelling was used to explore non-linear associations for the continuous variables of age and BMI. Associations of exposures and outcomes were determined on the basis of the magnitude of the point estimates. Examination of Schoenfeld residuals confirmed that the assumptions of proportionality were satisfied.

### Association of greenhouse gas emissions and land use with cancer

Incidence rates for all cancers and organ-specific cancers were assessed in relation to quartiles of greenhouse gas and land use in pooled and country-specific analyses. HRs were calculated with Cox proportional hazards regression models, where the underlying time scale was person-years from the start of the study until diagnosis, adjusting for the set of potential confounders, and additionally for country as a fixed effect in pooled models. Organ-specific cancer assessments were calculated separately for those cancers for which there was a sufficient number of cases (n>250) and include cancers of the brain and CNS, bladder, renal pelvis, ureter and other urinary organs, breast, cervix uteri, colorectum, endometrium, oesophagus, gallbladder and biliary tract, kidney, larynx, liver, lung, lymph nodes, myeloma, ovary, pancreas, prostate, skin melanoma, stomach, and thyroid.

### Counterfactual models for alternative diets

We investigated potential interventional effects of contrasting different dietary scenarios, using a counterfactual approach,^17^ in pooled analyses of all-cause mortality and all cancers. Dietary scenarios were derived by considering a diet that is potentially beneficial for both human health and environmental sustainability, based on ranges from the 14 key recommendations of the EAT–*Lancet* Commission.^10^ The construction of the EAT–*Lancet* diet score has been previously described elsewhere,^11^ and is provided in more detail for the present study in the appendix (pp 2–3, 5–7).

For time to event outcomes of all-cause mortality and cancer, adjusted Cox proportional hazards regression models were used to derive counterfactual probabilities to define the attributable fraction at specific timepoints ranging from 0 to 20 years.^17^ This approach estimates the proportion of outcome events that would be prevented before time (t) if the exposure was eliminated at baseline (t_0_). Models were adjusted for the set confounders described previously. Attributable fraction was an estimate of interest since it can indicate the public health impact of interventions for different dietary scenarios.

Models were first fit with the EAT–*Lancet* diet score as a continuous variable to estimate the standardised probabilities of survival and cancer rates for the score, and were then plotted. Additionally, we fit models setting EAT–*Lancet* diet scores to a few specified counterfactual values, based on both a population's potential ability to shift to specific EAT–*Lancet* scores as an intervention and the distribution of EAT–*Lancet* scores in the population (appendix p 22). Specifically, we computed the counterfactual probability of outcomes (Y), p(Y_x_=1), by setting x to scores of 3, 8, 9, 10, and 14, with 3 representing the lowest score in our dataset, 8 representing the mean, 9 representing the median, 10 representing the 75% percentile, and 14 representing the highest score possible (ie, perfect adherence), in separate models, compared to a hypothetical score of 0. We compared the scores to 0, as attributable fraction models assess the potential effects of an exposure on an outcome if the exposure was hypothetically eliminated from the population; in the present study this corresponds to no adherence to the EAT–*Lancet* diet scores. However, because no one in the study had a score of 0, we also compared EAT–*Lancet* diet scores of 8, 9, 10, and 14 to the minimum score found in EPIC of 3. From these predicted probabilities, counterfactual attributable fraction models^17^ were used to simulate the proportion of outcome events that would be prevented if dietary EAT–*Lancet* diet scores were hypothetically changed in the population, assuming a causal effect of diet on the outcomes.

### Role of the funding source

The funders of EPIC did not play a role in data collection, data analysis, data interpretation, writing of the manuscript, or the decision to submit for publication.

## Results

Of the 443 991 participants in the EPIC cohort, 314 852 (71%) were female and 129 139 (29%) were male, largely because France and Norway had female-only cohorts. The average age at recruitment was 52 years (range 18–99), with 29% of participants receiving only a primary school education or no education, and 80% were married or living together. 50% of individuals were former or current smokers and 50% never smoked, 53% were not physically active, and 52% were overweight or obese at recruitment (table 1). Country-specific demographic data are provided in the appendix (p 8).

**Table 1:** Demographic characteristics of the EPIC cohort

		**EPIC cohort (n=443 991)**
Age at recruitment (years)		52 (10; 18–99)
Sex		
	Female	314 852 (71%)
	Male	129 139 (29%)
Education		
	Not educated or primary school education only	127 204 (29%)
	Technical or professional school	103 452 (23%)
	High school	94 317 (21%)
	Higher education (university)	119 018 (27%)
Marital status		
	Not married	89 812 (20%)
	Married or living together	354 179 (80%)
Smoking status		
	Never smoker	220 583 (50%)
	Former smoker	123 319 (27%)
	Current smoker	100 089 (23%)
Physical activity		
	Not active	234 854 (53%)
	Active	209 137 (47%)
	BMI, kg/m^2^	25 (4; 10–78)
	Overweight or obese (≥25 kg/m^2^)	242 312 (52%)
	Not overweight or obese (<25 kg/m^2^)	227 393 (48%)
Greenhouse gases (kg CO_2_ equivalents per kg food per day)		6·0 (1·92; 0·68–30·10)
	First quartile	3·6 (0·62; 0·68–4·39)
	Second quartile	5·0 (0·32; 4·40–5·59)
	Third quartile	6·2 (0·38; 5·60–6·89)
	Fourth quartile	8·4 (1·40; 6·90–30·10)
Land use (m^2^ per year per kg food per day)		7·2 (2·72; 0·79–48·40)
	First quartile	4·2 (0·78; 0·79–5·29)
	Second quartile	6·1 (0·46; 5·30–6·89)
	Third quartile	7·7 (0·55; 6·90–8·79)
	Fourth quartile	10·9 (2·00; 8·80–48·40)
Incident cancers		
	No cancer	385 066 (87%)
	Any cancer event	58 925 (13%)
Vital status		
	Alive	397 355 (90%)
	Deceased	46 636 (10%)
	Person-years of mortality	17·4 (3·6; 0·0–22·8)

Data are means (SD; range) for continuous variables and n (%) for categorical variables. BMI=body-mass index.

The average level of greenhouse gas emissions was 6·0 kg CO_2_ equivalents per kg food per day and the average level of land use was 7·2 m^2^ per year per kg food per day (table 1). France and Denmark had the highest average value of greenhouse gas emissions (appendix p 20), and France and Italy had the highest average value of land use (appendix p 20). The food categories that contributed to the highest greenhouse gas emissions and land use levels were meat and meat products, followed by dairy products (appendix p 21).

There were 46 636 (10·5%) deaths from all causes. In the pooled analysis, comparing the fourth quartile to the first quartile, the adjusted HR for estimated all-cause mortality was 1·13 (95% CI 1·10–1·16) for greenhouse gas emissions and 1·18 (1·15–1·21) for land use (table 2). Country-specific results were similar to the pooled analysis (appendix p 10).

**Table 2:** Adjusted hazard ratios for all-cause and cause-specific mortality estimated for greenhouse gas emissions and land use contributions from diet modelled as quartiles

		**Events (%)**	Greenhouse gas emissions: adjusted hazard ratios*(95% CI)			Land use contributions; adjusted hazard ratios*(95% CI)		
			Second quartile†	Third quartile†	Fourth quartile†	Second quartile†	Third quartile†	Fourth quartile†
All-cause mortality		46 636 (10·5%)	0·96 (0·94–0·99)	1·02 (0·99–1·04)	1·13 (1·10–1·16)	0·99 (0·96–1·01)	1·05 (1·03–1·08)	1·18 (1·15–1·21)
Cause-specific mortality								
	Coronary heart disease mortality	4944 (1·1%)	0·88 (0·81–0·96)	1·06 (0·97–1·14)	1·19 (1·10–1·30)	1·003 (0·93–1·09)	1·12 (1·04–1·21)	1·38 (1·27–1·49)
	Cardiovascular disease mortality	6393 (1·4%)	0·99 (0·93–1·07)	1·03 (0·95–1·10)	1·19 (1·10–1·28)	0·97 (0·91–1·04)	1·04 (0·97–1·11)	1·18 (1·10–1·27)
	Respiratory disease mortality	2479 (0·6%)	0·89 (0·78–0·99)	0·95 (0·84–1·06)	1·02 (0·91–1·15)	0·89 (0·91–1·00)	1·02 (1·09–1·14)	1·09 (0·97–1·22)
	Cancer mortality	14 095 (3·2%)	1·03 (0·98–1·08)	1·11 (1·05–1·16)	1·16 (1·10–1·22)	1·06 (1·01–1·11)	1·14 (1·09–1·20)	1·21 (1·16–1·27)

* Models adjusted for age at recruitment, marital status, education, physical activity, smoking status, and body-mass index. Pooled analyses (all countries) were also adjusted for country.

† The first quartile is the reference value.

Cause-specific mortality was also assessed, accounting for competing risks. There were 4944 reported deaths from coronary heart disease. In the pooled analysis, comparing the fourth quartile to the first quartile, the estimated adjusted HR for mortality from coronary heart disease was 1·19 (95% CI 1·10–1·30) for greenhouse gas emissions and 1·38 (1·27–1·49) for land use (table 2). There were 6393 reported deaths from cardiovascular disease. In the pooled analysis, comparing the fourth quartile to the first quartile, the estimated HR for mortality from cardiovascular disease was 1·19 (95% CI 1·10–1·28) for greenhouse gas emissions and 1·18 (1·10–1·27) for land use (table 2). There were 2479 deaths related to respiratory disease, and these were positively associated with land use when comparing the third quartile to the first quartile (adjusted HR 1·02 [95% CI 1·09–1·14]). There were 14 095 deaths from cancer. In the pooled analysis, comparing the fourth quartile to the first quartile, the estimated HR for cancer mortality was 1·16 (95% CI 1·10–1·22) for greenhouse gas emissions and 1·21 (1·16–1·27) for land use. Results for cause-specific analyses stratified by each country varied in comparison with pooled analyses, but for most causes the number of deaths was low, resulting in imprecise estimates (appendix pp 12–15).

Concerning cancer incidence rates, the adjusted HR was 1·11 (95% CI 1·09–1·14) for all-cause cancer associated with greenhouse gas emissions and 1·13 (1·10–1·15) for all-cause cancer associated with land use, when comparing the fourth quartile to the first quartile (table 3). Results for analyses stratified by each country were similar to the results of pooled analyses (appendix p 16).

**Table 3:** Adjusted hazard ratios for all-cause and cancer-specific incidence rates estimated for greenhouse gas emissions and land use contributions from diet, modelled as quartiles

	**Events (%)**	Greenhouse gas emissions: adjusted hazard ratios*(95% CI)			Land use contributions: adjusted hazard ratios*(95% CI)		
		Second quartile†	Third quartile†	Fourth quartile†	Second quartile†	Third quartile†	Fourth quartile†
All-cause cancer	58 925 (12·9%)	1·03 (1·01–1·06)	1·08 (1·06–1·11)	1·11 (1·09– 1·14)	1·04 (1·01–1·06)	1·10 (1·07– 1·12)	1·13 (1·10–1·15)
Brain and CNS	827 (0·2%)	1·14 (0·93–1·39)	1·18 (0·97–1·44)	0·93 (0·76–1·15)	1·01 (0·83–1·24)	1·30 (1·06–1·56)	1·09 (0·89–1·33)
Bladder, renal, pelvis, ureter, and other urinary organs	1584 (0·4%)	1·17 (1·01–1·36)	1·29 (1·12–1·50)	1·47 (1·28–1·70)	1·20 (1·03–1·40)	1·45 (1·25–1·68)	1·52 (1·31–1·76)
Breast‡	13 283 (3·3%)	1·05 (1·01–1·10)	1·15 (1·10–1·21)	1·21 (1·15–1·27)	1·07 (1·02–1·12)	1·14 (1·09–1·20)	1·23 (1·17–1·29)
Cervical‡	350 (0·1%)	0·88 (0·68–1·14)	0·71 (0·53–0·95)	0·66 (0·48–0·91)	0·83 (0·64–1·09)	0·72 (0·54–0·97)	0·65 (0·47–0·90)
Colorectum	6141 (1·5%)	1·01 (0·94–1·09)	1·10 (1·02–1·18)	1·05 (0·98–1·13)	1·03 (0·96–1·11)	1·13 (1·05–1·22)	1·12 (1·04–1·20)
Endometrium‡	1925 (0·7%)	0·94 (0·84–1·06)	0·90 (0·80 1·02)	0·90 (0·79–1·03)	0·85 (0·76–0·96)	0·90 (0·80–1·02)	0·82 (0·72–0·94)
Oesophagus	468 (0·1%)	1·17 (0·85–1·59)	1·52 (1·14–2·04)	2·06 (1·55–2·72)	1·12 (0·82–1·52)	1·59 (1·20–2·12)	1·93 (1·46–2·55)
Gallbladder and biliary tract	335 (0·1%)	0·98 (0·73–1·34)	0·92 (0·67–1·25)	0·96 (0·71–1·30)	1·02 (0·75–1·39)	1·13 (0·84–1·53)	0·89 (0·64–1·23)
Kidney	1003 (0·3%)	1·14 (0·95–1·39)	1·18 (0·99–1·43)	1·43 (1·20–1·72)	1·07 (0·88–1·29)	1·26 (1·04–1·51)	1·46 (1·22–1·76)
Larynx	295 (0·1%)	1·07 (0·73–1·58)	1·51 (1·05–2·16)	1·70 (1·20–2·41)	1·19 (0·80–1·78)	1·47 (1·01–2·15)	2·17 (1·52–3·10)
Liver	439 (0·1%)	0·94 (0·70–1·25)	1·11 (0·84–1·46)	1·27 (0·97–1·66)	1·42 (1·06–1·90)	1·26 (0·94–1·70)	1·61 (1·21–2·14)
Lung	3777 (1·0%)	1·03 (0·93–1·13)	1·12 (1·02– 1·23)	1·15 (1·05– 1·27)	1·11 (1·01–1·23)	1·19 (1·08–1·31)	1·23 (1·11–1·35)
Lymph nodes	1397 (0·4%)	0·98 (0·84–1·14)	1·00 (0·86–1·16)	1·03 (0·89–1·20)	0·95 (0·82–1·10)	0·98 (0·84–1·13)	0·94 (0·81–1·10)
Myeloma	1833 (0·5%)	0·96 (0·84–1·10)	1·01 (0·88–1·15)	1·14 (1·00–1·30)	1·01 (0·88–1·16)	1·07 (0·94–1·23)	1·17 (1·03–1·34)
Ovary‡	1415 (0·5%)	1·04 (0·90–1·19)	1·08 (0·94–1·24)	0·93 (0·79–1·09)	1·03 (0·90–1·18)	1·00 (0·86–1·15)	0·85 (0·72–1·00)
Pancreas	1289 (0·3%)	0·96 (0·82–1·14)	1·14 (0·98–1·34)	1·17 (0·99–1·37)	1·08 (0·92–1·27)	1·14 (0·97–1·34)	1·25 (1·07–1·47)
Prostate§	6902 (6·0%)	1·04 (0·96–1·13)	1·02 (0·94–1·11)	1·05 (0·97–1·13)	1·16 (1·07–1·27)	1·15 (1·05–1·24)	1·17 (1·08–1·27)
Skin melanoma	4567 (1·2%)	1·11 (1·02–1·21)	1·16 (1·07–1·26)	1·13 (1·04–1·23)	1·10 (1·01–1·20)	1·13 (1·04–1·23)	1·10 (1·01–1·20)
Stomach	979 (0·2%)	1·02 (0·84–1·24)	1·21 (1·01–1·45)	1·27 (1·05–1·52)	1·16 (0·95–1·41)	1·33 (1·10–1·61)	1·52 (1·26–1·83)
Thyroid	757 (0·2**%**)	1·16 (0·93–1·44)	1·35 (1·09–1·66)	1·42 (1·15–1·75)	1·17 (0·94–1·47)	1·48 (1·20–1·83)	1·64 (1·33–2·02)

* Models adjusted for age at recruitment, marital status, education, physical activity, smoking status, and body-mass index.

† The first quartile is the reference value.

‡ Women only.

§ Men only.

For incidence rates of specific cancers there was a positive association between greenhouse gas emissions and cancers of the bladder, renal pelvis, ureter and other urinary organs, breast, colorectum, oesophagus, kidney, larynx, lung, skin melanoma, stomach, and thyroid. There was also a positive association between land use and cancers of the brain and CNS, bladder, renal pelvis, ureter and other urinary organs, breast, colorectum, oesophagus, kidney, larynx, liver, lung, myeloma, pancreas, prostate, skin melanoma, stomach, and thyroid (table 3).

**Figure 1:**
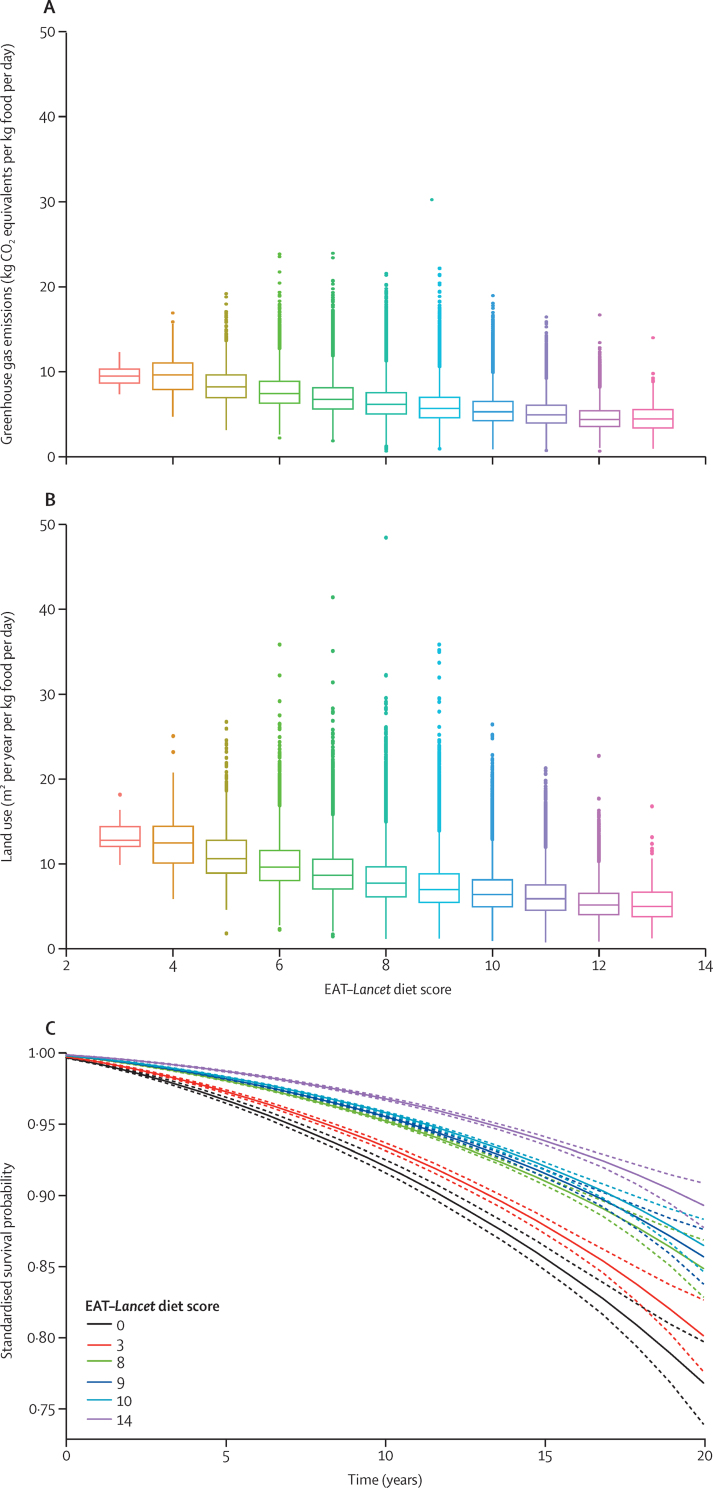
Levels of greenhouse gas emissions (A) and land use by EAT–*Lancet* diet scores (B), and adjusted estimated survival probabilities for different values of the EAT–*Lancet* diet score across a 20-year period (C)

The distribution of the EAT–*Lancet* diet score is shown in the appendix (p 22); the median value was 9, with a range of 3 to 13. In general, the higher the EAT–*Lancet* diet score was, the lower the mean greenhouse gas emissions and land use values were (figure 1A, B). Participants who had the highest score of 13 had a mean value of 5 kg CO_2_ equivalents per kg food per day for greenhouse gas emissions and 5 m^2^ per year per kg food per day for land use, whereas those who had the lowest score of 3 had a mean value of 10 kg CO_2_ equivalents per kg food per day for greenhouse gas emissions and 13 m^2^ per year per kg food per day for land use (figure 1A, B). Thus, shifting from a dietary score of 3 to 13 would result in a 50% reduction in greenhouse gas emissions and a 62% reduction in land use.

The calculated standardised survival probabilities were lower for lower values of the EAT–*Lancet* diet score, and higher for higher values, indicating better survival outcomes with a higher EAT–*Lancet* diet score (figure 1C). Assessing the attributable fraction for all-cause mortality using counterfactual values of 3, 8, 9, 10, and 14 compared to 0 indicated that increasing scores led to an increase in the attributable fraction, which decreased over time; however, a score of 0 compared to individuals' true factual score had an inverse attributable fraction (appendix p 23). When comparing an EAT–*Lancet* diet score of 3 (low adherence to the score) to a score of 0 (no adherence to the score), the attributable fraction decreased from 19% at year 0 to 14% at year 20. This suggests that 14–19% of deaths could have been prevented by adhering to an EAT–*Lancet* diet score of 3 versus not adhering to an EAT–*Lancet* diet at all in a 20-year period. Comparatively, the attributable fraction increased as the score increased: when comparing an EAT–*Lancet* diet score of 9 (the median level in the cohort) to a score of 0 (no adherence to the diet) the attributable fraction decreased from 47% at year 0 to 38% at year 20. When comparing a score of 14 (perfect adherence to the diet) to a score of 0, the attributable fraction decreased from 63% at year 0 to 54% at year 20, suggesting that 54–63% of deaths could be prevented in a 20-year period by fully adopting the EAT–*Lancet* diet. Similar results were observed when simulating a counterfactual EAT–*Lancet* diet score of 3 versus scores of 8, 9, 10, and 14, although the effects sizes were not as large in magnitude.

Assessing the attributable fractions for cancer incidence rates when comparing counterfactual values of 3, 8, 9, 10, and 14 to an EAT–*Lancet* diet score of 0 indicated that increasing scores led to an increase in the attributable fraction (appendix p 24). When comparing the EAT–*Lancet* diet score of 3 (minimum score in the cohort) to 0 (no adherence) the attributable fraction was 10% at year 0 and 8% at year 20, and when comparing an EAT–*Lancet* diet score of 14 (perfect adherence to the diet) to a score of 0 (no adherence to the diet), the attributable fraction decreased from 39% at year 0 to 35% at year 20. Taken together, 10–39% of cancers could be prevented by adopting the EAT–*Lancet* diet in a 20-year risk period. Similar trends were observed when comparing simulated dietary scores to the lowest value in the cohort, a counterfactual EAT–*Lancet* diet score of 3 (the minimum value in the cohort) versus scores of 8, 9, 10, and 14, although the effects were not of a sufficiently large magnitude.

Results from the sensitivity analyses are presented in the appendix (pp 17–19).

**Figure 2:**
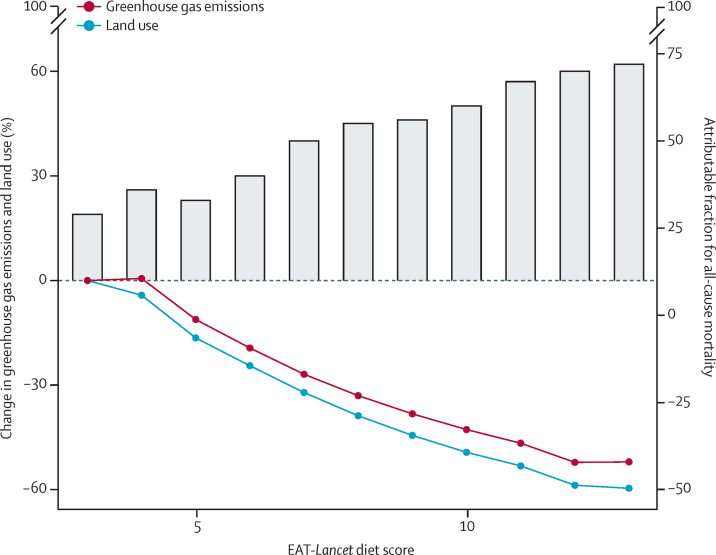
Co-benefits of the EAT–*Lancet* diet score Lines represent the proportion of greenhouse gas emissions and land use that would change with adherence to EAT–*Lancet* diet scores (compared to lower adherence: ie, a score of 3) and the bars represent the counterfactual attributable fraction from modelling shifts in diets and in deaths (ie, all-cause mortality) that could be prevented over a 20-year risk period from adhering to a higher score of the EAT–*Lancet* reference diet.

The co-benefits of shifting towards higher EAT–*Lancet* diet scores, representing a potential increase in the attributable fraction for all-cause mortality and a reduction in greenhouse gas emissions and land use, are shown in figure 2. Greenhouse gas emissions could be reduced up to 50% and land use levels reduced up to 62%, by eating foods that span a higher EAT–*Lancet* diet score compared to eating foods that comprise a lower EAT–*Lancet* diet score.

## Discussion

In the large prospective cohort of EPIC, with a follow-up of 14 years and more than 400 000 participants, we observed an association between levels of greenhouse gas emissions and land use, estimated from dietary components collected from food frequency question-naires, and all-cause and cause-specific mortality, and all-cause cancers and some organ-specific cancers. By simulating dietary interventions that consider nutritional needs and environmental footprints, based on the EAT–*Lancet* reference diet, all-cause mortality and cancer rates could be substantially reduced, alongside a potential reduction in greenhouse gas emissions and land use. These findings suggest that co-benefits for human health and the environment could be achieved by adhering to diets that consider both nutritional quality and the health of the planet.

We found an association with increasing levels of greenhouse gas emissions and land use estimated from dietary components and all-cause mortality. Similar findings were evident for cause-specific mortality in pooled assessments, with the largest HR for cancer mortality. Additionally, we found an association with levels of greenhouse gas emissions and land use estimated from dietary components with cancer rates in pooled assessments. The majority of previous studies investigating the associations between environmental footprints and mortality or morbidity, or both, did not quantify footprints derived from diet-based food frequency data, but instead focused on potential interventions and risk reductions through model-based assumptions of levels of land use and greenhouse gas emissions, among other assumptions (as reviewed by Quam and colleagues^18^), thus limiting comparison of our findings with the published literature. However, a previous study from the EPIC Netherlands cohort investigated the association between greenhouse gas emissions and land use (from what was considered to be the usual diet in that population) and all-cause or cause-specific mortality, and did not find any associations.^19^ Nonetheless, the authors did find, by modelling a one-third reduction in total meat consumption, a major contributor to dietary greenhouse gas emissions and land use, there was an inverse association with mortality. Several studies have investigated the links between specific foods (ie, those that contribute to higher greenhouse gas emissions and land use) and mortality or morbidity, or both. For example, there is a large body of evidence supporting the links between increased red meat consumption and morbidity and mortality,^20^ including an increased risk of type 2 diabetes,^21^ cardiovascular disease,^22^ certain types of cancer,^23^ and mortality.^24^

According to our counterfactual attributable fraction models, up to 63% of deaths and 39% of incident cancers could be prevented in a 20-year risk period by fully adopting the EAT–*Lancet* reference diet (ie, perfect adherence), compared to not adopting the diet. A reduction in mortality from adhering to the EAT–*Lancet* reference diet is supported by findings from the EAT–*Lancet* Commission report, where results from one analysis estimated that, by adopting their reference diet, about 11·1 million deaths per year could be avoided worldwide by 2030 and premature mortality reduced by 19%.^25^ Notably, these previous EAT–*Lancet* analyses were based on food availability data and not on individual consumption data, as in the present study. However, a replication study done in the USA did not support a reduction in mortality from adopting the EAT–*Lancet* diet, and the authors suggested the need for further independent validation.^26^ In other studies, alternative diets meeting both nutritional recommendations and greenhouse gas reductions led to substantial improvements in population health, including an increase in life expectancy and improvements in disability-adjusted life-years.^27^ Additionally, a global impact evaluation showed that reductions of up to 10% in premature mortality could be achieved mostly through a reduction in red meat consumption, leading to 80–90% of diet-related reductions in greenhouse gas emissions by 2050.^25^ In a previous study by the EPIC Oxford cohort, adhering to the EAT–*Lancet* reference diet, based on the same score used in the present study, was shown to be beneficial in terms of reducing rates of ischaemic heart disease and diabetes, as well as BMI, but there were no associations with stroke and mortality.^11^ A main difference between the present study and the study by Knuppel and colleagues,^11^ besides differences in sample size and the statistical approach, that could influence our mortality findings is that the EPIC Oxford cohort comprised a large number of vegetarians, thus influencing associations between altering diet and related outcomes, which is not consistent with the dietary makeup of all EPIC cohorts considered in the present study. Supporting the co-benefits of our findings, we also found that reductions in greenhouse gas emissions and land use could occur with higher adherence to the EAT–*Lancet* reference diet, whereby greenhouse gas emissions could be reduced by 50% and land use levels by 62%. Overall, adhering to the EAT–*Lancet* reference diet seems to be beneficial for reducing all-cause mortality and cancer while mutually reducing greenhouse gas emissions and land use; this is particularly concerning for Europe's local environmental impacts, where agricultural production is among the most intensive in the world.^28^

Our study had various limitations. Greenhouse gas emissions and land use estimates were based on self-reported country-specific dietary questionnaires that are, like any dietary intake assessment, prone to measurement error (eg, under-reporting) and potential misclassification. Additionally, diet was assessed at one time, and although it was validated in a follow-up study,^29^ repeated measures might better capture dietary patterns and lifetime greenhouse gas emissions and land use from foods consumed. Moreover, the levels of greenhouse gas emissions and land use were derived from foods based on the SHARP database, which does not capture country-specific estimates, despite being representative of European-wide estimates, which might have led to some exposure misclassification; however, this is likely to be non-differential. Differences in land use and greenhouse gas estimates differ by country and mode of production,^30^ and should be considered in future cohort studies. Additionally, our study addressed individual dietary contributions of greenhouse gas emissions and land use, and thus our counterfactual scenarios relied on personal dietary behaviour changes for higher adherence to the EAT–*Lancet* reference diet. To truly achieve reductions in greenhouse gas emissions and land use, among other environmental impacts, we will need to consider entire food systems, consisting of all inputs, such as the environment, people, processes, infrastructure, institutions, waste, and so on, including activities and actors that relate to the production, processing, distribution, preparation, and consumption of food. Additionally, we did not take into consideration other important factors affecting dietary choices, including social, ethical, economic, cultural, and food safety indicators related to diets,^31^ nor do our findings reflect the important and understudied relationship between environmental footprints and health outcomes in low-income countries. Such approaches have been done at the country level,^32^ but need to be addressed with population-level cohort data. For our attributable fraction estimates of the relationships between potential interventional effects from dietary shifts in the EAT–*Lancet* diet score and mortality and cancer, we assumed a causal relationship, which has many underlying assumptions, including the assumption that there was no residual confounding; however, there might be residual confounding based on unmeasured confounders or imperfectly measured confounders, which should be kept in mind when interpreting effect estimates. Last, we acknowledge that there has been considerable debate surrounding the EAT–*Lancet* reference diet; although it serves as a broad framework for dietary guidance in the present study, we acknowledge that the EAT–*Lancet* reference diet might not be accessible to and followed by all individuals, and it might need to be adapted to local contexts.^33^ There are several challenges associated with developing a global sustainable diet, and future studies will need to consider these challenges; however, guidance on what constitutes a healthy diet, in particular a diet that achieves co-benefits, remains crucial to public health.

Our study had several strengths. The EPIC cohort consists of a large European population of more than 400 000 people with a long follow-up, increasing the generalisability of our findings to that of similar populations. Unique to our study is the use of cohort data comprising food frequency details for dietary contributions to greenhouse gas emissions and land use. The dietary assessments are comprehensive, consisting of detailed dietary data and their contributions to greenhouse gas emissions and land use for a large amount of food items, which decreases the potential measurement error of greenhouse gas emissions and land use contributions from diets. By using the EAT–*Lancet* diet scores we consider dietary scenarios that are healthy alternatives in terms of both nutritional requirements and lower greenhouse gas emissions and land use. Our study overcomes limitations of previous studies for modelled sustainable dietary patterns that did not consider nutritional requirements, or that considered such patterns but did not consider planetary health,^3^ whereas the EAT–*Lancet* reference diet improves the intakes of most nutrients compared with other environmentally sustainable diets.^10^ Additionally, although we only considered greenhouse gas emissions and land use contributions, adopting the EAT–*Lancet* diet could be associated with several additional environmental benefits, as this diet was developed within the planetary boundaries framework that considers several environmental impacts (eg, water use, acidification, eutrophication, and loss of biodiversity) to define targets for sustainable food production.^10^ Moreover, dietary score patterns are assessed holistically instead of concentrating on one particular food item, which might not reflect achievable dietary changes (eg, adopting a vegetarian diet). Furthermore, we used counterfactual models, which might strengthen our causal interpretations and have been highlighted as an essential assessment for co-benefits.^34^

In conclusion, we found co-benefits in terms of both health outcomes and reduction of greenhouse gas emissions and land use from shifting to a universally sustainable diet. Our results support the need for continued efforts to reduce consumption of foods that contribute to higher levels of greenhouse gas emissions and land use for both human and planetary health. Addressing dietary patterns that represent co-benefits could help in climate change mitigation, in addition to reducing other sources of greenhouse gas emissions, along with meeting other planetary health goals, and help reduce the incidence of diet-related mortality and cancers.

## Data sharing

EPIC data and biospecimens are available for investigators who seek to answer important questions on health and disease in the context of research projects that are consistent with the legal and ethical standard practices of the International Agency for Research on Cancer (IARC), WHO, and the EPIC centres. The primary responsibility for accessing the data, obtained in the frame of the present publication, belongs to the EPIC centres that provided them. The use of a random sample of anonymised data from the EPIC study can be requested by contacting epic@iarc.fr. The request will then be passed on to members of the EPIC Steering Committee for deliberation and approval.

## Declaration of interests

MRB presented at two conferences, which may be a conflict of interest: the MAYOLY-SPINDLER Symposium on pancreatology in practice at the Journées Francophones d'Hépato-gastroentérologie et d'Oncologie Digestive e-conference in 2020 (Why do I see more and more pancreatic cancers?) and the GILEAD e-conference on weight gain and HIV infection in 2020. All other authors declare no competing interests.

## References

[1] Branca F, Lartey A, Oenema S (2019). Transforming the food system to fight non-communicable diseases. BMJ.

[2] Jaacks LM, Vandevijvere S, Pan A (2019). The obesity transition: stages of the global epidemic. Lancet Diabetes Endocrinol.

[3] Aleksandrowicz L, Green R, Joy EJ, Smith P, Haines A (2016). The impacts of dietary change on greenhouse gas emissions, land use, water use, and health: a systematic review. PLoS One.

[4] Perignon M, Vieux F, Soler LG, Masset G, Darmon N (2017). Improving diet sustainability through evolution of food choices: review of epidemiological studies on the environmental impact of diets. Nutr Rev.

[5] Payne CL, Scarborough P, Cobiac L (2016). Do low-carbon-emission diets lead to higher nutritional quality and positive health outcomes? A systematic review of the literature. Public Health Nutr.

[6] Hyland JJ, Henchion M, McCarthy M, McCarthy SN (2017). The role of meat in strategies to achieve a sustainable diet lower in greenhouse gas emissions: a review. Meat Sci.

[7] Pairotti MB, Cerutti AK, Martini F, Vesce E, Padovan D, Beltramo R (2015). Energy consumption and GHG emission of the Mediterranean diet: a systemic assessment using a hybrid LCA-IO method. J Clean Prod.

[8] Tilman D, Clark M (2014). Global diets link environmental sustainability and human health. Nature.

[9] González-García S, Esteve-Llorens X, Moreira MT, Feijoo G (2018). Carbon footprint and nutritional quality of different human dietary choices. Sci Total Environ.

[10] Willett W, Rockström J, Loken B (2019). Food in the Anthropocene: the EAT–*Lancet* Commission on healthy diets from sustainable food systems. Lancet.

[11] Knuppel A, Papier K, Key TJ, Travis RC (2019). EAT-*Lancet* score and major health outcomes: the EPIC-Oxford study. Lancet.

[12] Riboli E, Hunt KJ, Slimani N (2002). European Prospective Investigation into Cancer and Nutrition (EPIC): study populations and data collection. Public Health Nutr.

[13] van Buuren S, Groothuis-Oudshoorn K (2011). mice: multivariate imputation by chained equations in R. J Stat Softw.

[14] Mertens E, Kaptijn G, Kuijsten A, van Zanten H, Geleijnse JM, van 't Veer P (2019). SHARP-Indicators Database towards a public database for environmental sustainability. Data Brief.

[15] European Food Safety Authority (EFSA) (2015). The food classification and description system FoodEx 2 (revision 2). EFSA Supporting Publications.

[16] Lau B, Cole SR, Gange SJ (2009). Competing risk regression models for epidemiologic data. Am J Epidemiol.

[17] Sjölander A (2018). Estimation of causal effect measures with the R-package stdReg. Eur J Epidemiol.

[18] Quam VGM, Rocklöv J, Quam MBM, Lucas RAI (2017). Assessing greenhouse gas emissions and health co-benefits: a structured review of lifestyle-related climate change mitigation strategies. Int J Environ Res Public Health.

[19] Biesbroek S, Bueno-de-Mesquita HB, Peeters PH (2014). Reducing our environmental footprint and improving our health: greenhouse gas emission and land use of usual diet and mortality in EPIC-NL: a prospective cohort study. Environ Health.

[20] Händel MN, Cardoso I, Rasmussen KM (2019). Processed meat intake and chronic disease morbidity and mortality: an overview of systematic reviews and meta-analyses. PLoS One.

[21] Pan A, Sun Q, Bernstein AM (2011). Red meat consumption and risk of type 2 diabetes: 3 cohorts of US adults and an updated meta-analysis. Am J Clin Nutr.

[22] Micha R, Wallace SK, Mozaffarian D (2010). Red and processed meat consumption and risk of incident coronary heart disease, stroke, and diabetes mellitus: a systematic review and meta-analysis. Circulation.

[23] Vieira AR, Abar L, Chan DSM (2017). Foods and beverages and colorectal cancer risk: a systematic review and meta-analysis of cohort studies, an update of the evidence of the WCRF-AICR Continuous Update Project. Ann Oncol.

[24] Larsson SC, Orsini N (2014). Red meat and processed meat consumption and all-cause mortality: a meta-analysis. Am J Epidemiol.

[25] Springmann M, Wiebe K, Mason-D'Croz D, Sulser TB, Rayner M, Scarborough P (2018). Health and nutritional aspects of sustainable diet strategies and their association with environmental impacts: a global modelling analysis with country-level detail. Lancet Planet Health.

[26] Zagmutt FJ, Pouzou JG, Costard S (2020). The EAT–*Lancet* Commission's dietary composition may not prevent noncommunicable disease mortality. J Nutr.

[27] Cobiac LJ, Scarborough P (2019). Modelling the health co-benefits of sustainable diets in the UK, France, Finland, Italy and Sweden. Eur J Clin Nutr.

[28] Bais-Moleman AL, Schulp CJE, Verburg PH (2019). Assessing the environmental impacts of production- and consumption-side measures in sustainable agriculture intensification in the European Union. Geoderma.

[29] Kroke A, Klipstein-Grobusch K, Voss S (1999). Validation of a self-administered food-frequency questionnaire administered in the European Prospective Investigation into Cancer and Nutrition (EPIC) Study: comparison of energy, protein, and macronutrient intakes estimated with the doubly labeled water, urinary nitrogen, and repeated 24-h dietary recall methods. Am J Clin Nutr.

[30] Poore J, Nemecek T (2018). Reducing food's environmental impacts through producers and consumers. Science.

[31] Mertens E, Van't Veer P, Hiddink GJ, Steijns JM, Kuijsten A (2017). Operationalising the health aspects of sustainable diets: a review. Public Health Nutr.

[32] Chaudhary A, Krishna V (2019). Country-specific sustainable diets using optimization algorithm. Environ Sci Technol.

[33] Steenson S, Buttriss JL (2020). The challenges of defining a healthy and ‘sustainable’ diet. Nutrition Bulletin.

[34] Haines A (2017). Health co-benefits of climate action. Lancet Planet Health.

