# Supplementary material for: Co-benefits from sustainable dietary shifts for population and environmental health: an assessment from a large European cohort study
Source: Lancet Planet Health. 2021 Oct 22;5(11):e786–96. doi: 10.1016/S2542-5196(21)00250-3 (PMC8581185; doi:10.1016/S2542-5196(21)00250-3)
Supplement: Supplementary appendix [file mmc1.pdf]

THE LANCET
Planetary Health

Supplementary appendix

This appendix formed part of the original submission and has been peer reviewed. We post it as supplied by the authors.

Supplement to: Laine JE, Huybrechts I, Gunter MJ, et al. Co-benefits from sustainable dietary shifts for population and environmental health: an assessment from a large European cohort study. *Lancet Planet Health* 2021; published online Oct 21. http://dx.doi.org/10.1016/S2542-5196(21)00250-3.

Co-benefits from sustainable dietary shifts for population and environmental health: an assessment from a large European cohort study

Appendix: Supplementary Information

Supplementary Methods:

Cohort Description

All participants in EPIC provided written informed consent and the ethical review boards from the International Agency for Research on Cancer (IARC) and all local centres approved the study. In most centres, dietary questionnaires (DQs) were self-administered, with the exception of Ragusa (Italy), Naples (Italy) and Spain, where face-to-face interviews were performed. Extensive quantitative DQs were used in northern Italy, the Netherlands, and Germany that were structured by meals in Spain, France and Ragusa. Semi-quantitative food-frequency questionnaires (FFQs) were used in Denmark, Norway, Naples, Umeå (Sweden) and the United Kingdom, while an FFQ was combined with a 7-day record on hot meals in Malmö (Sweden).[1] Post-harmonisation of all the questionnaire data was done by following standardized procedures (e.g. decomposing recipes and complex foods into ingredients) to obtain a standardised food list for which the level of detail is comparable between countries.

Data on vital status were obtained from mortality registries, in combination with data collected through active follow-up and next-of-kin. The end of follow-up/closure dates of the study period varied between 2009 and 2014 depending on the countries. Cause-specific mortality data were coded according to the 10th revision of the International Statistical Classification of Diseases, Injuries and Causes of Death (ICD-10). Causes of death assessed include, coronary heart disease (CHD) (ICD-10 codes: I20–I25), cardiovascular disease (CVD) (ICD-10 codes:I00–I99 excluding I20–I25), cancer (for alcohol-related cancer (ARC), including colorectal cancer (C18–C20), female breast cancer (C50), upper aerodigestive cancers (UADT, including cancer of the mouth (C01–C10 without C08=salivary gland), larynx (C21), pharynx (C11–C14), oesophagus (C15)), and respiratory disease (ICD-10 codes: J00–J99). Incident cancer cases were identified through several methods, including record linkage with population-based cancer registries, health insurance records, pathology registries, autopsy or death certificate, and active follow-up of study subjects. First primary invasive cancers were considered as cases in this study. Main cancer cases were coded according to the International Classification of Diseases for Oncology (ICD-O).[2]

For the present study, participants with prevalent or past cancer at baseline (n=25,184), and with missing information on dietary information (n=6,259), follow-up information (n=4,148) and in the highest and lowest 1% of the distribution for the ratio energy intake to estimated energy requirement (n=9,573) were excluded from the analysis. Additionally, those whose vital status was unknown either because they withdrew from the study, emigrated to another region, emigrated to another country, or

other unknown reasons were not included in the present study (n= 6,455). Participants from Greece were excluded due to administrative reasons.

Greenhouse Gas emission and Land Use Calculations from Food Frequency Data

Most food items from EPIC DQs were matched exactly by their FoodEx2 codes; however, for 1,985 (16.7%) items we used a proxy as the exact match was not available in the SHARP database. There were 298 (2.5%) food items for which we did not have a match and were not included in our GHG or LU estimates; though, most of these food items were rarely consumed, and thus would have negligible impact on our analyses. In the SHARP database, GHG emissions were expressed in kilograms of carbon dioxide equivalents per kilogram of food as eaten per day (kg CO_2eq per kg food per day) and LU as meters^2 per year per kilogram of food as eaten per day (m^2 per year per kg food per day). These GHG emission and LU values were used to derive levels of individual daily GHG emissions and LU for each participant.

Causal Structure and Confounding Variables

All information, on potential confounders, except for BMI, were collected at baseline via questionnaires. BMI was derived from weight and height measured in all centres, except for Oxford, France, and Norway where these were self- reported. Assessed weight and height were used to calculate BMI defined as weight in kilograms divided by height in metres squared (kg/m^2). Sex was treated as a potential confounder and assessed as an effect modifier. We considered sex separately from the other potential confounders for the following reasons: while sex is often a confounder or modifier of diet and outcomes assessed in the present study, it us unknown if sex would influence the consumption of GHG- and LU-related foods. Additionally, because the sampling frame differed by country in relation to sex, where there were 70% more females than males in the pooled data, we assessed potential sex-based effects by assessing potential confounding by sex compared to the crude model in those cohorts with both sexes represented (thus excluding France and Norway). We determined that sex was not a potential confounder based on the small changes in estimates.

Counterfactual Models for Alternative Diets

The EAT-Lancet Diet Score has been previously described elsewhere[3]; however, we expand the description here. The score was constructed considering possible ranges for the EAT Lancet diet recommendations (as displayed in Table S1).[4] Specifically, the values for the EAT-Lancet Diet Score were chosen using the minimum value where there was a suggested range that did not include zero and the maximum value for any foods where the possible range included zero. These values were

chosen from the ranges suggested by the EAT-Lancet reference diet to allow for a range of values possible and to reflect the median values of macronutrient intake that the authors used in constructing the EAT-Lancet reference diet. Additionally, a higher level within the range was not used to prevent over-estimates for following the diet, and to not impose an ideal setting to the data, which was not expected by the EAT Lancet commission's description of the universal reference diet.

To construct the EAT-Lancet Diet Score, participants were assigned a point for meeting each of the recommendations for 14 dietary categories of 1) whole grains, 2) tubers and starchy vegetables, 3) vegetables, 4) fruits, 5) dairy foods, protein sources of 6) beef, lamb, pork, 7) chicken or other poultry, 8) eggs, 9) fish, legumes of 10) beans, lentils and peas, 11) soy foods, and 12) peanuts or tree nuts, 13) added fats, and 14) added sugars. Scores were tallied, resulting in possible scores ranging from 0 to 14, with 14 representing perfect adherence. A higher EAT-Lancet Diet Score consists of higher consumption of vegetables, fruits, nuts, and unsaturated oils, and includes none to low/moderate amounts of proteins including, legumes, eggs, soy foods, seafood, poultry, red meat, processed meat, added sugars, refined grains, and starchy vegetables.

Of the 11,000 unique food items there were 9,138 captured in the EAT-Lancet Diet Score.

Sensitivity Analysis

As a sensitivity analysis, pooled models were assessed accounting for random effects of the country where the EPIC center was located, using mixed effects Cox models with the R package coxme.[3] As another sensitivity analysis our main models for the outcomes of cause-specific and all-cause mortality and all cancers were adjusted for energy intake as it may be a potential confounder. We consider this as a sensitivity analysis and not as one of our main confounders as the role of energy intake as a confounder is not clear, where previous co-benefits analyses found that correction for energy did not alter estimates.[4] Energy intake was derived from the DQs, but matching with country-specific food composition tables according to standardized procedures.[5] Finally, because BMI may be on the causal path between exposure to GHG and LU foods and the outcomes, we ran additional sensitivity analyses not adjusting for BMI.

There was little discrepancy between fixed effects and random effects models. For all outcomes, the mixed effects models had nearly the same estimates for GHG and LU as the fixed effect models and the confidence intervals remained narrow (Table S9). The random center effect, an estimated intercept for each center, had varying standard deviations, with cause-specific mortalities having the highest (Table S9). This suggests there may be random effects from the different centers. Adjusting for energy intake reduced the estimates of GHG for all-cause mortality, and cause-specific mortality for CHD, CVD, cancer, respiratory disease, but not cancer rates; however, this was not true for most LU estimates which remained significant (Table S10). Not including BMI as a variable in the model influenced the estimates, mostly increasing the effects, for all-cause mortality, CHD mortality,

and CVD mortality but not for others; (Table S11) however, it may be important to run more robust mediation models, such as counterfactual mediation models in future studies.

Country Specific Results

Because EPIC is a multicentre cohort, designed for harmonisation, our main results and conclusions are drawn from pooled estimates. However, it is important to discuss country specific results that may differ from our main conclusions. For the assessments between dietary contributions and all-cause mortality we found an association in every country, except for France. This could be due to under-reporting, potential residual confounding of lifestyle and/or behaviours unique to this population, the selection of the population as they are all women of higher education, or the so-called French paradox or simply there is no association.

Table S1 Construction of the EAT-LANCET diet score based on previous defined+ overarching dietary categories and food frequency items from EPIC

**Dietary category**	**Criteria for scoring 1 point**	**Specific food items from food frequency questionnaire**	**Number of food items from food frequency questionnaire**
**Whole grains**	<=464g/day and whole grain fibre >5 grams	Cereal and cereal products; Cereal product non-specified or combined; Flour, flakes, starches, semolina; Pasta, rice, other grains; Pasta, rice, other grains non-specified or combined; Other grains (100% cereal); Pasta-like cereal-based products (not 100% cereal); Bread, crispbread, rusks; Bread, crispbread, rusks non-specified or combined; Crispbread, rusks; Breakfast cereals; Salty biscuits, aperitif biscuits, crackers; Dough and pastry (puff, short-crust, pizza); Dough, pastry non-specified or combined; Bread/pizza dough	926
**Tubers and starchy vegetables**	<=100g/day	Potatoes and other tubers; Potatoes and other tubers non-specified or combined	181
**Vegetables**	>=200g/day	Carrots, spinach, broccoli, leafy greens, brussels sprouts, cabbage, peas, green beans, courgettes, cauliflower, parsnips, leeks, onion, garlic, mushrooms, sweet peppers, beansprouts, salad vegetables, watercress, tomatoes, sweetcorn, beetroot, coleslaw, avocado; Vegetables non-specified; Leafy vegetables; Fruiting vegetables; Root vegetables; Cabbages;	2432

		Mushrooms; Onion, garlic; Stalk vegetables, sprouts; Mixed salad; Mixed vegetables	
**Fruits**	>=100g/day	Fruit peel; Fruit non-specified; Citrus fruits; Apple and pear; Grape; Stone fruits; Other fruits; Berries; Banana; Kiwi; Non citrus fruits non-specified (only for Spain); Mixed fruits	631
**Dairy foods Whole milk or derivative equivalents**	<=500g/day	Dairy products; Dairy product non-specified or combined; Milk; Milk beverages; Yoghurt, thick fermented milk; Curd; Cheese; Cheese non-specified or combined; Ricotta; Other cheeses; Cream desserts, puddings (milk based); Dairy creams; Milk for coffee and creamers (dairy)	1079
**Beef, lamb, pork**	<=28g/day	Beef, Veal; Pork; Mutton/lamb	687
**Chicken, other poultry**	<=58g/day	Poultry; Poultry non-specified or combined; Chicken, hen; Turkey; Duck; Goose	208
**Eggs**	<=25g/day	Eggs; Egg products	201
**Seafood**	<=100g/day	Fish and shellfish; Fish and shellfish non-specified or combined; Fish; Fish non-specified or combined<br>Lean (white) fish; Fatty/ very fatty fish; Crustaceans, molluscs; Fish products, fish in crumbs; Fish products non-specified or combined; Lean fish products; Fatty fish products; roe; Fish liver	987
**Legumes**	<=100g/day	Legumes non-specified or combined; Legumes	90
**Soy foods**	<=50g/day	Soya products	72
**Nuts**	>=25g/day	Nuts (-spread) and seeds; Nuts, seeds non-specified or combined; Tree nuts; Peanuts; Seeds	89

**Added fats**	Ratio of 0.8 for unsaturated to saturated fat intake	Fat; Fat non-specified or combined; Vegetable oils; Oil non-specified; Olive oil; Soya oil; Sunflower oil; Peanut oil; Corn/maize oil; G rape oil; Rapeseed oil; Safflower oil; Walnut oil; Other oil; Mixed oil; Butter; Margarine; Margarine, origin non-specified; Margarine, pure vegetable; Margarine, mixed origin; Deep frying fats; Marine oils; Other animal fat	1038
**Added sugars and desserts**	<=31g/day	Sugar, honey, jam non-specified or combined; Sugars; Honey; Jam/syrup; Chocolate, candy bars, paste, confetti; Confectionery non-chocolate, candied fruits; Ice cream, water ice; Ice cream, water ice non-specified or combined; Ice cream; Sorbet, water ice; Cakes and biscuits; Cakes, biscuits non-specified or combined; Cakes, sweet pies, pastries, puddings (non-milk based); Dry cakes, biscuit; Artificial sweeteners	517
**Total**			9138

[+] Knuppel A, Papier K, Key TJ, Travis RC. EAT-Lancet score and major health outcomes: the EPIC-Oxford study. Lancet. 2019;394(10194):213-214.

Supplementary Results:

Table S2 Demographic characteristics for the EPIC cohorts in the present study presented by country

	Denmark (54,664)	France (67,358)	Germany (47,938)	Italy (43,786)	Nether-lands (35,652)	Norway (33,793)	Spain (39,902)	Sweden (47,250)	UK (73,648)
**Age at recruitment (years)**	57 (50-66)	53 (42-71)	51 (20-70)	51 (24-78)	50 (20- 70)	48 (41-56)	49 (29-70)	52 (29-74)	50 (17.8- 98.5)
**Sex**									
Female	28,594 (52)	67,358 (100)	27,011 (56)	30,068 (69)	26,420 (74)	33,793 (100)	24,797 (62)	25,613 (54)	51,198 (70)
Male	26070 (48)	-	20927 (44)	13718 (31)	9232 (26)	-	15105 (38)	21637 (46)	22450 (30)
**Education**									
Not educated/ Primary school	18053 (33)	8009 (12)	11292 (24)	21591 (49)	5733 (16)	7746 (23)	29301 (73)	16844 (36)	8635 (12)
Technical/ Professional school	20949 (38)	278 (0.4)	17072 (36)	5369 (12)	12654 (36)	12082 (35)	3340 (8)	11453 (24)	20255 (28)
High school	5381 (10)	34147 (51)	3268 (7)	10880 (25)	9965 (28)	9739 (29)	2611 (7)	8764 (19)	9562 (13)
Higher education of university	10281 (19)	24924 (37)	16306 (34)	5946 (14)	7300 (20)	4226 (13)	4650 (12)	10189 (21)	35196 (47)
**Marital status**									
Not married	10472 (19)	11563 (17)	10719 (22)	6496 (15)	10579 (30)	519 (2)	7093 (18)	12184 (26)	20187 (27)
Married or living together	44192 (81)	55795 (83)	37219 (78)	37290 (85)	25073 (70)	33274 (98)	32809 (82)	35066 (74)	53461 (73)
**Smoking Status**									
Never Smoker	19229 (35)	47315 (70)	21978 (46)	19909 (45)	13651 (38)	12292 (36)	22151 (55)	23124 (49)	40934 (56)
Former Smoker	16655 (31)	13733 (20)	15953 (33)	11753 (26)	11182 (31)	10457 (31)	7046 (18)	12963 (27)	23577 (32)
Current Smoker	18780 (34)	6310 (10)	10007 (21)	12124 (29)	10819 (30)	11044 (33)	10705 (27)	11163 (24)	9137 (12)
**Physical Activity**									
Not Active	22725 (42)	39548 (59)	25547 (53)	29578 (68)	10384 (29)	8450 (25)	28556 (72)	26740 (57)	43326 (59)
Active	31939 (58)	27810 (41)	22391 (47)	14208 (32)	25268 (71)	25343 (75)	11346 (28)	20510 (43)	30322 (41)
**BMI**	22 (13-59)	23 (13- 58)	26 (13-59)	26 (15-67)	25 (13-58)	24 (13-55)	28 (16-66)	25 (10-78)	25 (13-75)
**Cancer**									
Non-Cancer	43338 (89)	60228 (89)	43336 (90)	38749 (88)	31371 (88)	30197 (89)	35424 (89)	38264 (81)	63970 (87)
Any Cancer Event	11326 (21)	7130 (11)	4602 (10)	5037 (12)	4281 (12)	3596 (11)	4478 (11)	8986 (19)	9678 (13)
**Person Years Cancer Rates**	14.9 (0.01-19.1)	12.9 (0.1-15.4)	10.5 (0.005-15.5)	14.3 (0.005-18.6)	14.4 (0.01-18.0)	13.3 (0.03-14.1)	16.0 (0.01-20.4	16.7 (0.003-23.0)	15.0 (0.003-19.9)
**Vital Status**									
Alive	45246 (83)	62,924 (93)	44515 (93)	41363 (94)	32181 (90)	32647 (97)	36474 (91)	38632 (82)	63373 (86)
Deceased	9418 (17)	4434 (7)	3423 (7)	2423 (6)	3471 (10)	1146 (3)	3428 (9)	8618 (18)	10275 (14)
**Person Years Mortality**	16.2 (0.1-19.6)	19.2	13.8 (0 -20.7)	15.6 (0.2-20.9)	16.9 (0.1-20)	13.0	19 (0.1-21.5)	18.3 (0.1-22.8)	16.8 (0.1 -20.9)

		(0.1-20.7)				(0.1-14.1)			

Table S3 Adjusted Hazard Ratios (HR) and 95% Confidence Intervals (CI) for all-cause mortality and all-cause cancer estimated for levels of greenhouse gases (GHG) and land use (LU) contributions from diet modelled as quartiles

	GHG Quartiles			LU quartiles		
	2nd	3rd	4th	2nd	3rd	4th
	HR (95% CI)			HR (95% CI)		
All countries	0.96 (0.94, 0.99)	1.02 (0.99, 1.04)	1.13 (1.10, 1.16)	0.99 (0.96, 1.01)	1.05 (1.03, 1.08)	1.18 (1.15, 1.21)
Denmark	1.01 (0.93, 1.09)	1.06 (1.01, 1.14)	1.23 (1.15, 1.32)	1.03 (0.95, 1.12)	1.10 (1.02, 1.19)	1.29 (1.19, 1.39)
France	0.97 (0.88, 1.08)	0.91 (0.82, 1.00)	0.95 (0.87, 1.05)	0.90 (0.81, 1.00)	0.91 (0.83, 1.01)	0.94 (0.86, 1.04)
Germany	0.94 (0.85, 1.04)	1.16 (1.05, 1.28)	1.25 (1.14, 1.37)	1.03 (0.93, 1.14)	1.17 (1.06, 1.30)	1.41 (1.28, 1.55)
Italy	0.96 (0.86, 1.09)	1.08 (0.96, 1.2)	1.10 (0.97, 1.23)	0.91 (0.79, 1.04)	1.01 (0.89, 1.20)	1.04 (0.91, 1.18)
Netherlands	1.07 (0.92, 1.10)	1.02 (0.93, 1.11)	1.15 (1.04, 1.28)	0.97 (0.88, 1.07)	1.07 (0.97, 1.20)	1.16 (1.04, 1.29)
Norway	0.88 (0.77, 1.01)	1.02 (0.86, 1.20)	1.06 (0.80, 1.10)	0.95 (0.83, 1.08)	1.09 (0.90, 1.30)	1.03 (0.64, 1.65)
Spain	0.96 (0.87, 1.06)	1.01 (0.92, 1.12)	1.10 (1.01, 1.20)	1.02 (0.92, 1.12)	1.04 (0.94, 1.14)	1.11 (1.01, 1.22)
Sweden	0.94 (0.89, 0.99)	1.00 (0.95, 1.06)	1.14 (1.07, 1.21)	1.00 (0.93, 1.05)	1.04 (0.98, 1.10)	1.27 (1.19, 1.35)
United Kingdom	0.94 (0.89, 1.00)	0.97 (0.91 1.02)	1.03 (0.98, 1.10)	0.99 (0.94, 1.04)	1.04 (0.98, 1.10)	1.10 (1.03, 1.15)

Table S4 Adjusted+ Hazard Ratios (HR) and 95% Confidence Intervals (CI) for all-cause and cause-specific mortality estimated for levels of greenhouse gases (GHG) and land use (LU) contributions from diet modelled quartiles for all participants (n=443,991)

	N (%) Events	GHG Quartiles*			LU Quartiles*		
		2nd	3rd	4th	2nd	3rd	4th
		HR (95% CI)			HR (95% CI)		
**All-cause**							
	46,636 (10.5)	0.96 (0.94, 0.99)	1.02 (0.99, 1.04)	1.13 (1.10, 1.16)	0.99 (0.96, 1.01)	1.05 (1.03, 1.08)	1.18 (1.15, 1.21)
**CHD**							
	4,944 (1.1)	0.88 (0.81, 0.96)	1.06 (0.97, 1.14)	1.19 (1.10, 1.30)	1.003 (0.93, 1.09)	1.12 (1.04, 1.21)	1.38 (1.27, 1.49)
**CVD**							
	6,393 (1.4)	0.99 (0.93, 1.07)	1.03 (0.95, 1.10)	1.19 (1.10, 1.28)	0.97 (0.91, 1.04)	1.04 (0.97, 1.11)	1.18 (1.10, 1.27)
**Respiratory**							
	2,479 (0.6)	0.89 (0.78, 0.99)	0.95 (0.84, 1.06)	1.02 (0.91, 1.15)	0.89 (0.91, 1.00)	1.02 (1.09, 1.14)	1.09 (0.97, 1.22)
**Cancer**							
	14,095 (3.2)	1.03 (0.98, 1.08)	1.11 (1.05, 1.16)	1.16 (1.10, 1.22)	1.06 (1.007, 1.11)	1.14 (1.09, 1.20)	1.21 (1.16, 1.27)

+Models adjusted for age at recruitment, marital status, education, physical activity, smoking status, and BMI.
*the 1st quartile is the referent value.

Table S5 Adjusted+ Hazard Ratios (HR) and 95% Confidence Intervals (CI) for coronary heart disease (CHD) mortality estimated for levels of greenhouse gases (GHG) and land use (LU) contributions from diet modelled quartiles

	N (%) Events	GHG Quartiles*			LU Quartiles*		
		2nd	3rd	4th	2nd	3rd	4th
		CHD HR (95% CI)			CHD HR (95% CI)		
All	4944 (1.1)	0.88 (0.81, 0.96)	1.05 (0.97, 1.14)	1.19 (1.10, 1.29)	1.003 (0.93, 1.09)	1.12 (1.04, 1.21)	1.38 (1.27, 1.49)
Denmark	777 (1.4)	0.99 (0.74, 1.32)	1.29 (0.98, 1.69)	1.50 (1.15, 1.95)	1.01 (0.73, 1.40)	1.49 (1.10, 2.02)	1.80 (1.34, 2.42)
France	107 (0.16)	0.56 (0.29, 1.09)	0.72 (0.41, 1.29)	0.85 (0.50, 1.46)	1.00 (0.50, 2.02)	1.04 (0.54, 2.00)	1.19 (0.64, 2.21)
Germany	446 (0.93)	0.85 (0.63, 1.14)	1.25 (0.95, 1.65)	1.47 (1.13, 1.91)	0.92 (0.68, 1.24)	1.47 (1.11, 1.95)	1.72 (1.30, 2.26)
Italy	154 (0.35)	0.89 (0.56, 1.44)	0.96 (0.60, 1.53)	0.95 (0.60, 1.50)	0.73 (0.42, 1.27)	0.91 (0.55, 1.52)	0.86 (0.52, 1.41)
Netherlands	269 (0.75)	0.64 (0.45, 0.92)	1.03 (0.75, 1.43)	1.40 (0.98, 1.99)	0.66 (0.45, 0.96)	0.96 (0.68, 1.36)	1.51 (1.06, 2.16)
Norway	62 (0.18)	0.91 (0.51, 1.63)	0.88 (0.41, 1.86)	1.48 (0.57, 3.85)	1.00 (0.57, 1.76)	1.02 (0.47, 2.24)	1.63 (0.39, 6.89)
Spain	309 (0.77)	0.91 (0.65, 1.27)	0.91 (0.65, 1.27)	1.09 (0.80, 1.49)	1.09 (0.78, 1.52)	0.89 (0.63, 1.26)	1.14 (0.83, 1.58)
Sweden	1377 (3.0)	0.89 (0.77, 1.02)	0.98 (0.85, 1.14)	1.31 (1.13, 1.52)	1.01 (0.88, 1.17)	1.09 (0.94, 1.26)	1.62 (1.38, 1.89)
United Kingdom	1443 (2.0)	0.96 (0.82, 1.13)	1.06 (0.91, 1.24)	1.00 (0.85, 1.17)	1.21 (1.05, 1.39)	1.06 (0.91, 1.23)	1.17 (1.00, 1.36)

+Models adjusted for age at recruitment, marital status, education, physical activity, smoking status, and BMI.
*the 1st quartile is the referent value.

Table S6 Adjusted+ Hazard Ratios (HR) and 95% Confidence Intervals (CI) for cardiovascular disease (CVD) mortality estimated for levels of greenhouse gases (GHG) and land use (LU) contributions from diet modelled quartiles

	N (%) Events	GHG Quartiles*			LU Quartiles*		
		2nd	3rd	4th	2nd	3rd	4th
		CVD HR (95% CI)			CVD HR (95% CI)		
All	6,393 (1.4)	0.99 (0.93, 1.07)	1.03 (0.95, 1.10)	1.19 (1.10, 1.28)	0.97 (0.91, 1.04)	1.04 (0.97, 1.11)	1.18 (1.10, 1.27)
Denmark	1167 (2.1)	0.99 (0.79, 1.24)	1.09 (0.88, 1.35)	1.36 (1.10, 1.67)	0.95 (0.75, 1.19)	0.98 (0.79, 1.23)	1.28 (1.03, 1.59)
France	394 (0.58)	0.84 (0.60, 1.16)	0.76 (0.56 1.04)	0.87 (0.65, 1.17)	0.85 (0.60, 1.21)	0.91 (0.66, 1.26)	0.96 0.70, 1.30)
Germany	439 (0.92)	0.79 (0.59, 1.06)	1.13 (0.86, 1.49)	1.36 (1.05, 1.76)	0.91 (0.69, 1.22)	1.17 (0.89, 1.55)	1.47 (1.12, 1.93)
Italy	376 (0.86)	1.04 (0.77, 1.42)	1.16 (0.86, 1.58)	1.58 (1.18, 2.11)	0.95 (0.67, 1.36)	1.13 (0.81, 1.59)	1.40 (1.01, 1.94)
Netherlands	518 (1.5)	1.06 (0.84, 1.33)	0.97 (0.76, 1.24)	1.01 (0.76, 1.36)	1.09 (0.85, 1.39)	1.08 (0.84, 1.39)	1.06 (0.80, 1.41)
Norway	91 (0.27)	1.00 (0.63, 1.60)	1.18 (0.67, 2.07)	0.24 (0.033, 1.73)	1.12 (0.71, 1.76)	1.18 (0.62, 2.23)	2.65 (0.02, 3.00)
Spain	439 (1.1)	0.95 (0.73, 1.24)	0.88 (0.67, 1.16)	0.98 (0.75, 1.28)	0.98 (0.76, 1.28)	0.88 (0.67, 1.15)	1.02 (0.78, 1.34)
Sweden	1388 (3.0)	1.19 (1.03, 1.37)	1.21 (1.04, 1.40)	1.28 (1.09, 1.51)	1.15 (1.00, 1.33)	1.28 (1.11, 1.48)	1.40 (1.18, 1.66)
United Kingdom	1581 (2.1)	0.90 (0.78, 1.05)	0.91 (0.79, 1.05)	1.03 (0.89, 1.19)	0.87 (0.76, 1.00)	1.00 (0.88, 1.15)	1.01 (0.88, 1.17)

+Models adjusted for age at recruitment, marital status, education, physical activity, smoking status, and BMI.
*the 1st quartile is the referent value.

Table S7 Adjusted+ Hazard Ratios (HR) and 95% Confidence Intervals (CI) for respiratory disease mortality estimated for levels of greenhouse gases (GHG) and land use (LU) contributions from diet modelled quartiles

	N (%) Events	GHG Quartiles*			LU Quartiles*		
		2nd	3rd	4th	2nd	3rd	4th
		Respiratory deaths HR (95% CI)			Respiratory deaths HR (95% CI)		
All	2,479 (0.6)	0.89 (0.78, 0.99)	0.95 (0.84, 1.06)	1.02 (0.91, 1.15)	0.89 (0.91, 1.00)	1.02 (1.09, 1.14)	1.09 (0.97, 1.22)
Denmark	663 (1.2)	0.76 (0.58, 0.99)	0.87 (0.67, 1.11)	0.94 (0.74, 1.21)	0.93 (0.70, 1.24)	0.88 (0.67, 1.15)	1.03 (0.79, 1.35)
France	103 (0.15)	0.94 (0.48, 1.83)	1.03 (0.55, 1.93)	1.10 (0.60, 2.01)	0.88 (0.44, 1.76)	0.80 (0.41, 1.56)	1.28 (0.70, 2.34)
Germany	134 (0.28)	0.68 (0.40, 1.17)	1.26 (0.78, 2.04)	1.45 (0.91, 2.30)	0.93 (0.56, 1.56)	1.42 (0.87, 2.33)	1.61 (0.98, 2.65)
Italy	70 (0.16)	1.20 (0.63, 2.29)	0.99 (0.50, 1.96)	0.97 (0.48, 1.96)	1.09 (0.51, 2.31)	1.18 (0.57, 2.45)	0.83 (0.39, 1.79)
Netherlands	191 (0.54)	0.96 (0.64, 1.43)	1.13 (0.76, 1.69)	1.71 (1.11, 2.64)	0.95 (0.61, 1.46)	1.50 (0.99, 2.25)	1.63 (1.03, 2.56)
Norway	0						
Spain	158 (0.40)	1.05 (0.66, 1.65)	1.00 (0.63, 1.57)	0.88 (0.56, 1.39)	1.11 (0.70, 1.76)	1.07 (0.68, 1.69)	0.85 (0.53, 1.37)
Sweden	454 (0.96)	1.08 (0.84, 1.38)	1.18 (0.91, 1.52)	1.28 (0.97, 1.69)	0.98 (0.77, 1.26)	1.22 (0.95, 1.56)	1.35 (1.01, 1.79)
United Kingdom	706 (0.96)	0.81 (0.65, 1.00)	0.78 (0.63, 0.96)	0.83 (0.67, 1.02)	0.75 (0.61, 0.92)	0.94 (0.77, 1.16)	0.98 (0.79, 1.21)

+Models adjusted for age at recruitment, marital status, education, physical activity, smoking status, and BMI.
*the 1st quartile is the referent value.

Table S8 Adjusted+ Hazard Ratios (HR) and 95% Confidence Intervals (CI) for cancer mortality estimated for levels of greenhouse gases (GHG) and land use (LU) contributions from diet modelled quartiles

	N (%) events	GHG Quartiles*			LU Quartiles*		
		2nd	3rd	4th	2nd	3rd	4th
		Cancer deaths HR (95% CI)			Cancer deaths HR (95% CI)		
All	14095 (3.2)	1.03 (0.98, 1.08)	1.11 (1.05, 1.16)	1.16 (1.10, 1.22)	1.06 (1.007, 1.11)	1.14 (1.09, 1.20)	1.21 (1.16, 1.27)
Denmark	3,172 (5.8)	1.07 (0.93, 1.23)	1.18 (1.04, 1.35)	1.28 (1.12, 1.45)	1.06 (0.92, 1.22)	1.21 (1.06, 1.39)	1.31 (1.14, 1.50)
France	1,335 (2.0)	1.14 (0.94, 1.38)	1.07 (0.90, 1.29)	1.05 (0.88, 1.25)	0.99 (0.81, 1.21)	1.05 (0.87, 1.26)	1.01 (0.85, 1.21)
Germany	1,138 (2.4)	1.08 (0.92, 1.28)	1.12 (0.94, 1.33)	1.15 (0.97, 1.36)	1.16 (0.98, 1.38)	1.14 (0.95, 1.36)	1.30 (1.09, 1.55)
Italy	989 (2.3)	0.95 (0.78, 1.15)	1.11 (0.92, 1.33)	1.12 (0.94, 1.35)	1.00 (0.80, 1.25)	1.06 (0.86, 1.31)	1.12 (0.91, 1.38)
Netherlands	1,081 (3.0)	1.10 (0.93, 1.31)	1.09 (0.92, 1.30)	1.27 (1.04, 1.54)	0.99 (0.82, 1.18)	1.10 (0.92, 1.31)	1.26 (1.04, 1.53)
Norway	455 (1.3)	0.89 (0.72, 1.11)	1.05 (0.81, 1.37)	1.24 (0.82, 1.89)	1.00 (0.82, 1.24)	1.18 (0.88, 1.57)	1.20 (0.59, 2.43)
Spain	1,176 (3.0)	0.90 (0.76, 1.07)	1.00 (0.85, 1.18)	0.99 (0.84, 1.16)	1.07 (0.90, 1.28)	1.14 (0.96, 1.35)	1.13 (0.95, 1.34)
Sweden	2,440 (5.2)	1.08 (0.98, 1.21)	1.22 (1.09, 1.36)	1.26 (1.11, 1.42)	1.07 (0.96, 1.18)	1.19 (1.07, 1.33)	1.31 (1.16, 1.48)
United Kingdom	2,309 (3.1)	0.97 (0.86, 1.10)	1.03 (0.91, 1.16)	1.11 (0.98, 1.25)	1.06 (0.95, 1.19)	1.08 (0.96, 1.21)	1.13 (1.00, 1.28)

+Models adjusted for age at recruitment, marital status, education, physical activity, smoking status, and BMI.
*the 1st quartile is the referent value.

Table S9 Adjusted+ Hazard Ratios (HR) and 95% Confidence Intervals (CI) for cancer rates estimated for levels of greenhouse gases (GHG) and land use (LU) contributions from diet modelled as quartiles by country

	N (%) Events	GHG Quartiles*			LU Quartiles*		
		2nd	3rd	4th	2nd	3rd	4th
		Cancer HR (95% CI)			Cancer HR (95% CI)		
All	58,925 (12.9)	1.03 (1.01, 1.06)	1.08 (1.06, 1.11)	1.11 (1.09, 1.14)	1.04 (1.01, 1.06)	1.10 (1.07, 1.12)	1.13 (1.10, 1.15)
Denmark	11326 (20.7)	1.06 (0.99, 1.14)	1.13 (1.06, 1.21)	1.15 (1.08, 1.23)	1.06 (0.99, 1.15)	1.12 (1.05, 1.21)	1.18 (1.10, 1.27)
France	7130 (10.6)	1.08 (0.98, 1.18)	1.12 (1.03, 1.22)	1.11 (1.02, 1.20)	1.03 (0.94, 1.13)	1.09 (1.0004, 1.18)	1.07 (0.98, 1.16)
Germany	4602 (9.6)	1.08 (0.99, 1.17)	1.13 (1.04, 1.23)	1.05 (0.97, 1.15)	1.12 (1.03, 1.22)	1.12 (1.03, 1.22)	1.11 (1.02, 1.21)
Italy	5037 (11.5)	1.08 (0.99, 1.17)	1.07 (0.99, 1.17)	1.10 (1.02, 1.20)	1.01 (0.91, 1.12)	1.06 (0.96, 1.17)	1.12 (1.02, 1.23)
Netherlands	4281 (12.0)	1.02 (0.94, 1.11)	1.06 (0.97, 1.16)	1.04 (0.94, 1.15)	1.05 (0.97, 1.14)	1.08 (0.99, 1.18)	1.03 (0.94, 1.14)
Norway	3596 (10.6)	0.90 (0.84, 0.97)	0.92 (0.83, 1.01)	0.96 (0.81, 1.14)	0.91 (0.84, 0.97)	0.94 (0.84, 1.05)	0.87 (0.65, 1.17)
Spain	4478 (11.2)	1.01 (0.93, 1.10)	1.18 (1.08, 1.28)	1.18 (1.09, 1.29)	1.15 (1.05, 1.26)	1.23 (1.15, 1.37)	1.27 (1.16, 1.38)
Sweden	8986 (19.0)	1.04 (0.98, 1.10)	1.08 (1.02, 1.15)	1.21 (1.14, 1.30)	1.05 (0.99, 1.10)	1.11 (1.05, 1.17)	1.26 (1.19, 1.35)
United Kingdom	9678 (13.1)	1.04 (0.98, 1.10)	1.03 (0.98, 1.10)	1.08 (1.02, 1.15)	1.03 (0.98, 1.09)	1.10 (1.02, 1.14)	1.06 (1.00, 1.12)

+Models adjusted for age at recruitment, marital status, education, physical activity, smoking status, and BMI.
*the 1st quartile is the referent value.

Table S10 Sensitivity analyses assessing the random effect of country. Adjusted Hazard Ratios (HR) and 95% Confidence Intervals (CI) for all-cause mortality, coronary heart disease (CHD) mortality, cardiovascular disease (CVD) mortality, cancer mortality, respiratory disease mortality and cancer rates estimated for levels of greenhouse gases (GHG) and land use (LU) contributions from diet modelled as quartiles by country

	Standard Deviation (Variance) For Mixed Effect of Centre	GHG Quartiles*			LU Quartiles*		
		2nd	3rd	4th	2nd	3rd	4th
		HR /.(95% CI)					
All-cause mortality	0.25 (0.06)	0.96 (0.93, 0.98)	1.01 (0.99, 1.04)	1.12 (1.09, 1.15)	0.98 (0.96, 1.01)	1.05 (1.02, 1.08)	1.17 (1.14, 1.21)
CHD mortality	0.66 (0.44)	0.88 (0.81, 0.95)	1.05 (0.98, 1.14)	1.18 (1.09, 1.28)	1.00 (0.93, 1.08)	1.12 (1.04, 1.21)	1.37 (1.27, 1.49)
CVD mortality	0.36 (0.13)	0.97 (0.91, 1.04)	1.00 (0.94, 1.07)	1.16 (1.08, 1.24)	0.97 (0.91, 1.04)	1.03 (0.97, 1.11)	1.18 (1.10, 1.27)
Respiratory disease mortality	1.01 (1.03)	0.89 (0.80, 0.99)	0.95 (0.85, 1.07)	1.02 (0.91, 1.14)	0.89 (0.80, 0.99)	1.02 (0.91, 1.14)	1.09 (0.98, 1.23)
Cancer mortality	0.19 (0.03)	1.04 (0.99, 1.10)	1.12 (1.06, 1.17)	1.17 (1.11, 1.23)	1.06 (1.01, 1.11)	1.14 (1.08, 1.20)	1.21 (1.15, 1.28)
Cancer rates	0.25 (0.06)	1.04 (1.01, 1.06)	1.08 (1.05, 1.11)	1.11 (1.09, 1.14)	1.04 (1.02, 1.07)	1.10 (1.07, 1.12)	1.13 (1.10, 1.16)

*the 1st quartile is the referent value.

Table S11 Sensitivity analyses adjusting for energy intake. Adjusted Hazard Ratios (HR) and 95% Confidence Intervals (CI) for all-cause mortality, coronary heart disease (CHD) mortality, cardiovascular disease (CVD) mortality, cancer mortality, respiratory disease mortality and cancer rates for levels of greenhouse gases (GHG) and land use (LU) contributions from diet modelled as quartiles by country

	GHG Quartiles*			LU Quartiles*		
	2nd	3rd	4th	2nd	3rd	4th
	HR (95% CI)					
All-cause mortality	0.91 (0.88, 0.93)	0.91 (0.89, 0.94)	0.93 (0.90, 0.97)	0.94 (0.92, 0.97)	0.97 (0.94, 1.002)	1.03 (0.99, 1.07)
CHD mortality	0.82 (0.75, 0.89)	0.92 (0.84, 1.01)	0.93 (0.83, 1.05)	0.98 (0.90, 1.07)	1.05 (0.96, 1.16)	1.27 (1.12, 1.41)
CVD mortality	0.93 (0.86, 1.007)	0.91 (0.84, 0.99)	0.96 (0.86, 1.06)	0.92 (0.86, 1.00)	0.95 (0.88, 1.03)	1.00 (0.90, 1.11)
Respiratory disease mortality	0.85 (0.75, 0.96)	0.88 (0.77, 1.00)	0.89 (0.75, 1.05)	0.87 (0.77, 0.99)	0.99 (0.87, 1.13)	1.05 (0.89, 1.23)
Cancer mortality	0.99 (0.94, 1.04)	1.02 (0.97, 1.08)	1.04 (0.94, 1.07)	1.01 (0.96, 1.07)	1.06 (1.00, 1.13)	1.08 (1.01, 1.16)
Cancer rates	1.02 (1.01, 1.05)	1.06 (1.03, 1.08)	1.06 (1.02, 1.10)	1.03 (1.004, 1.05)	1.07 (1.04, 1.10)	1.09 (1.05, 1.13)

*the 1st quartile is the referent value.

Table S12 Sensitivity analyses not adjusting for BMI. Adjusted Hazard Ratios (HR) and 95% Confidence Intervals (CI) for all-cause mortality, coronary heart disease (CHD) mortality, cardiovascular disease (CVD) mortality, cancer mortality, respiratory disease mortality and cancer rates for levels of greenhouse gases (GHG) and land use (LU) contributions from diet modelled as quartiles by country

	GHG Quartiles*			LU Quartiles*		
	2nd	3rd	4th	2nd	3rd	4th
	HR (95% CI)					
All-cause mortality	0.97 (0.94, 0.99)	1.03 (1.00, 1.05)	1.15 (1.12, 1.18)	0.99 (0.97, 1.02)	1.07 (1.04, 1.10)	1.21 (1.17, 1.24)
CHD mortality	0.90 (0.82, 0.98)	1.08 (0.99, 1.17)	1.25 (1.15, 1.35)	1.03 (0.95, 1.12)	1.15 (1.06, 1.25)	1.48 (1.36, 1.60)
CVD mortality	1.005 (0.93, 1.08)	1.04 (0.97, 1.12)	1.22 (1.14, 1.32)	0.99 (0.92, 1.06)	1.08 (1.005, 1.16)	1.25 (1.16, 1.34)
Respiratory disease mortality	0.88 (0.78, 0.99)	0.94 (0.84, 1.06)	1.008 (0.90, 1.13)	0.88 (0.79 0.99)	1.012 (0.90, 1.14)	1.09 (0.96,1.22)
Cancer mortality	1.03 (0.98, 1.09)	1.11 (1.06, 1.17)	1.17 (1.11, 1.23)	1.05 (0.99, 1.10)	1.14 (1.08, 1.20)	1.21 (1.15, 1.28)
Cancer rates	1.04 (1.01, 1.06)	1.09 (1.06, 1.11)	1.12 (1.10, 1.15)	1.04 (1.02, 1.07)	1.10 (1.07, 1.13)	1.14 (1.11, 1.16)

*the 1st quartile is the referent value.

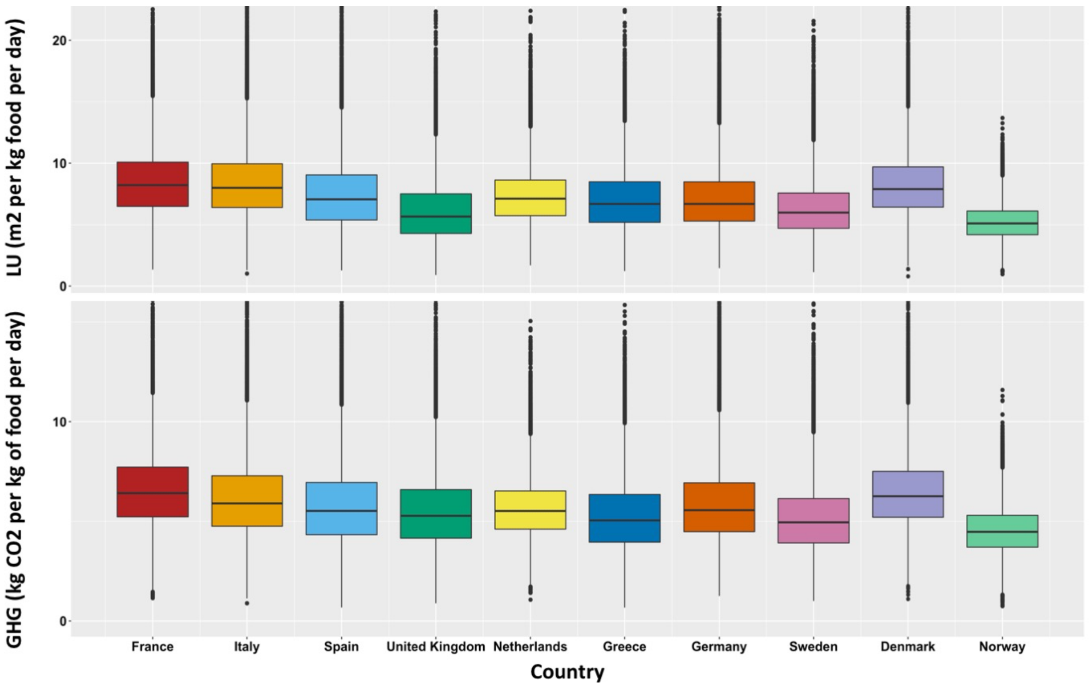

Figure S1 Levels of Greenhouse gases (GHG) and Land Use (LU) by country

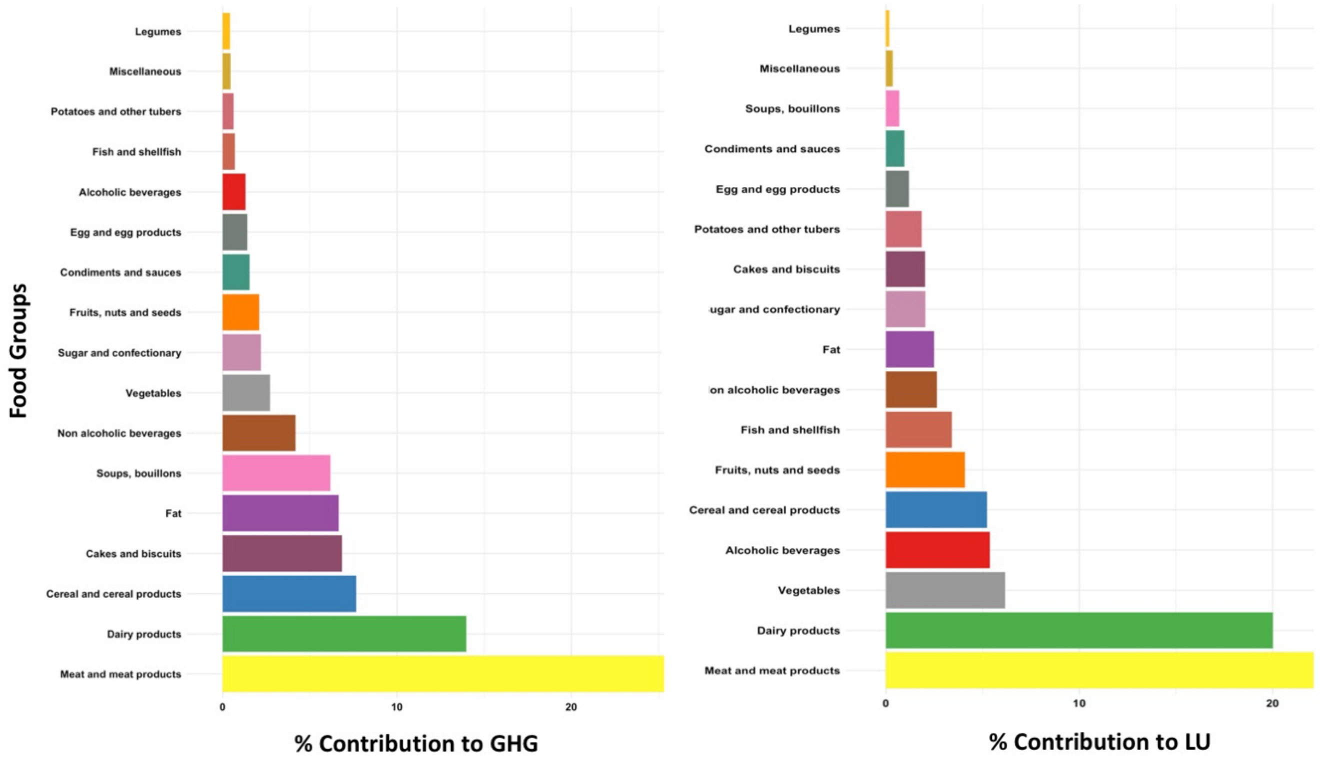

Figure S2 Levels of Greenhouse gases (GHG) and Land Use (LU) by overarching food groups

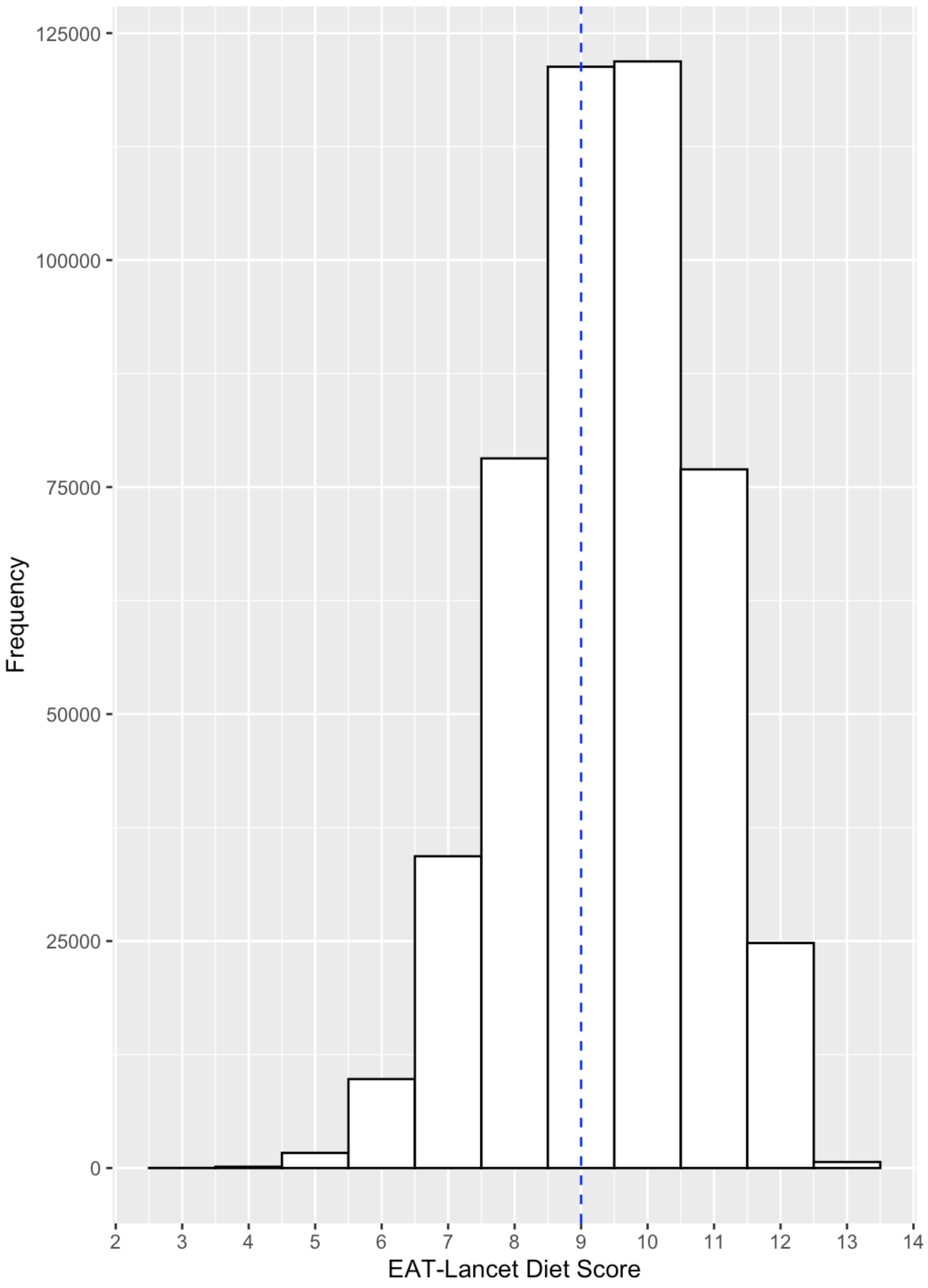

Figure S3 Distribution of EAT-Lancet Diet Scores in the EPIC cohort

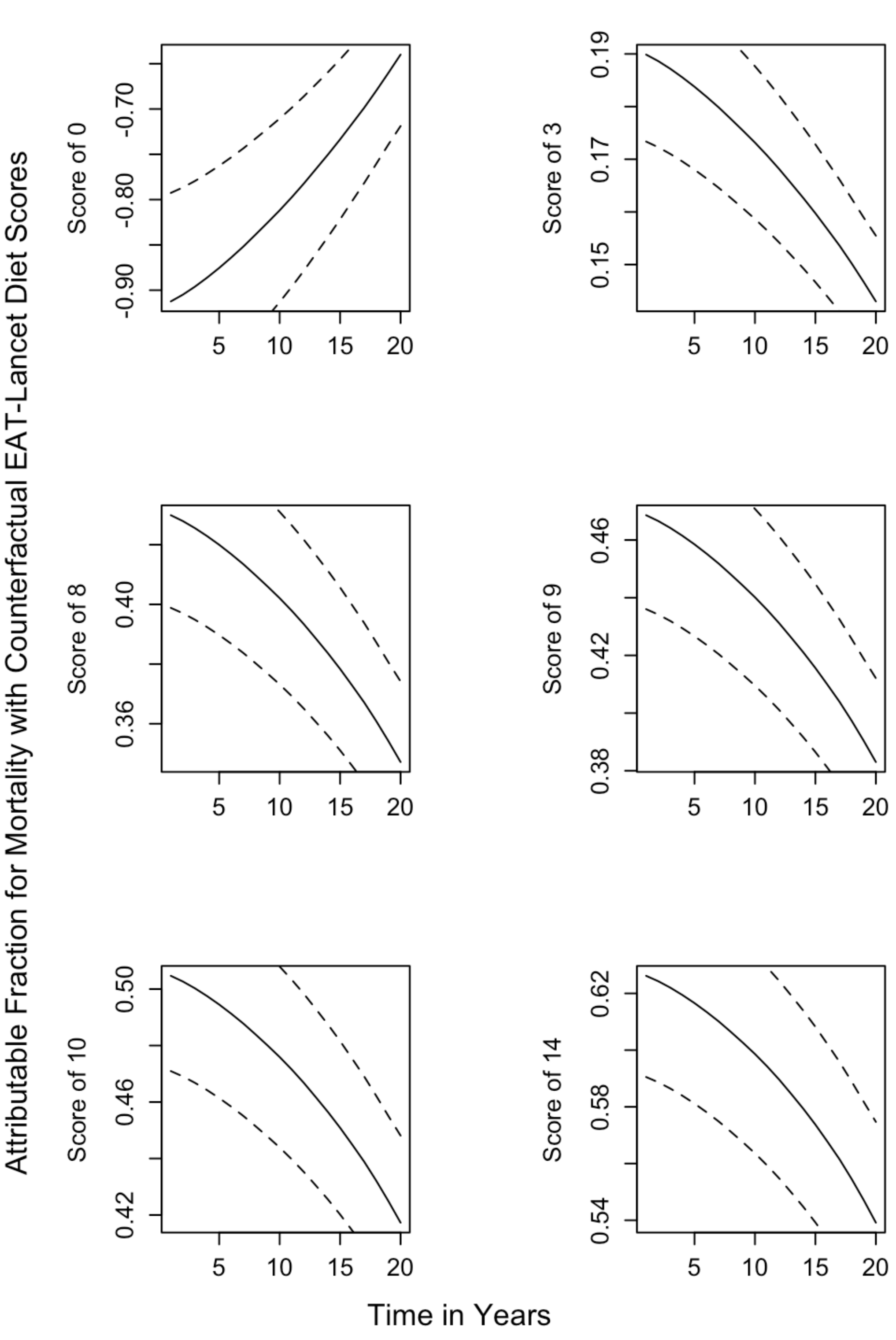

Figure S4. Counterfactual Attributable Fraction (AF) and 95% Confidence Intervals (CI) for all-cause mortality rates from adopting an EAT-Lancet Dietary Score of hypothetical values of 0 to the true factual mean score (of 9), and hypothetical scores of 3, 8, 9, 10, 14 compared to not adhering to an EAT-Lancet diet (a hypothetical score 0)

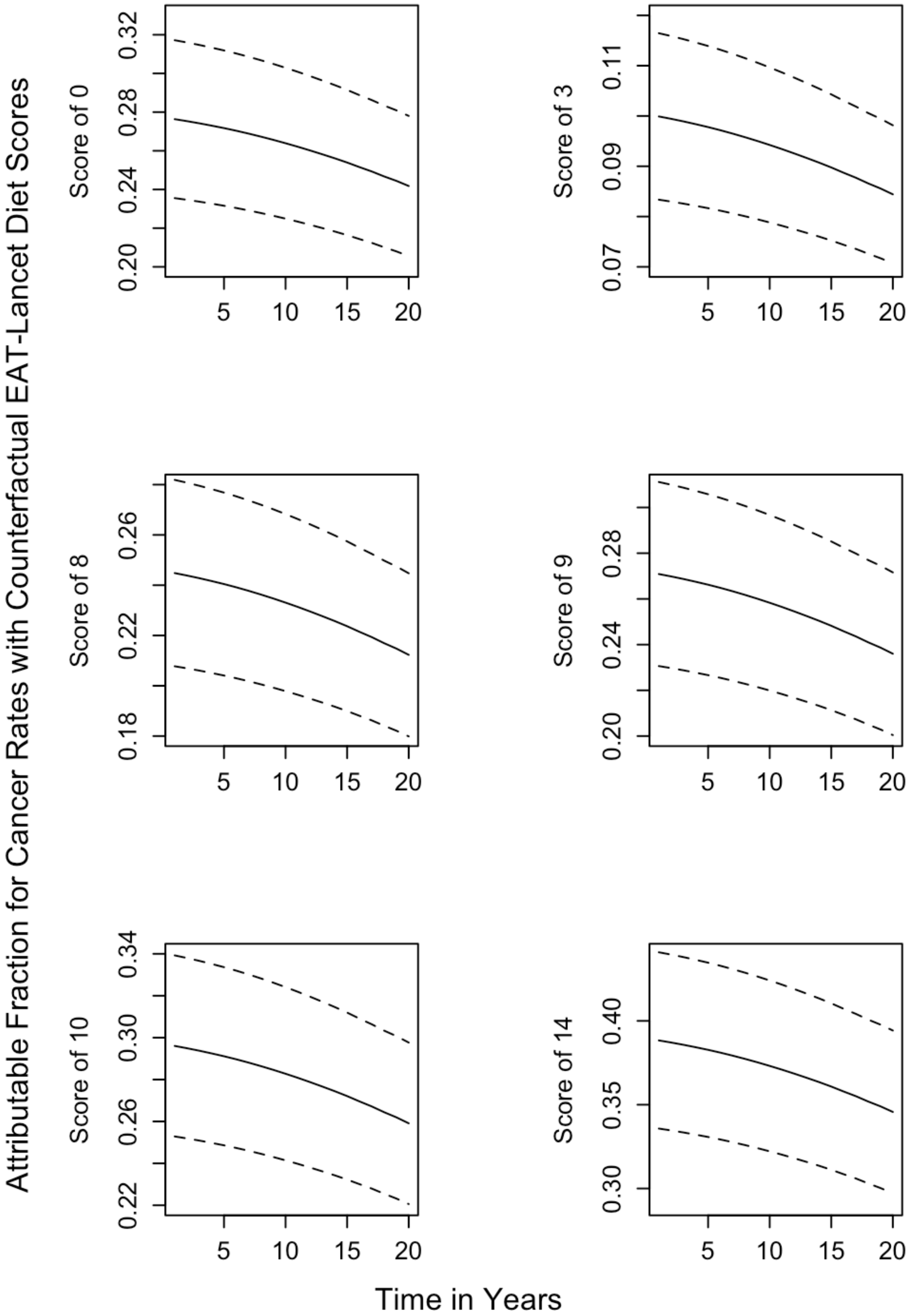

Figure S5. Counterfactual Attributable Fraction (AF) and 95% Confidence Intervals (CI) for all cancer rates from adopting an EAT-Lancet Dietary Score of hypothetical values of 0 to the true factual mean score (of 9), and hypothetical scores of 3, 8, 9, 10, 14 compared to not adhering to an EAT-Lancet diet (a hypothetical score 0)

REFERENCES

1. Riboli E, Hunt KJ, Slimani N, et al. European Prospective Investigation into Cancer and Nutrition (EPIC): study populations and data collection. Public health nutrition. 2002;5(6b):1113-1124.
2. International Classification of Diseases for Oncology, Third Edition, First Revision. Geneva: World Health Organization;2013.
3. Therneau TM. coxme: Mixed Effects Cox Models. In. R package version 2.2-162020.
4. Biesbroek S, Bueno-de-Mesquita HB, Peeters PH, et al. Reducing our environmental footprint and improving our health: greenhouse gas emission and land use of usual diet and mortality in EPIC-NL: a prospective cohort study. Environ Health. 2014;13(1):27.
5. Slimani N, Deharveng G, Unwin I, et al. The EPIC nutrient database project (ENDB): a first attempt to standardize nutrient databases across the 10 European countries participating in the EPIC study. Eur J Clin Nutr. 2007;61(9):1037-1056.
